# Graphdiyne as a Hole‐Transport Channel in Carbon Nitride Heterojunctions for Synergistic CO_2_ Reduction and *γ*‐Butyrolactone Synthesis

**DOI:** 10.1002/anie.3147768

**Published:** 2026-05-17

**Authors:** Xuan Zhang, Junqi Chai, Chong Wang, Wei Lin, Jing Tang, Oleksandr Savateev, Jimmy C. Yu, Jiajia Cheng

**Affiliations:** ^1^ State Key Laboratory of Chemistry for NBC Hazards Protection State Key Laboratory of Photocatalysis on Energy and Environment Sino‐UK International Joint Laboratory on Photocatalysis for Clean Energy and Advanced Chemicals & Materials College of Chemistry Fuzhou University Fuzhou P. R. China; ^2^ Department of Chemistry The Chinese University of Hong Kong Shatin Hong Kong SAR P. R. China; ^3^ Laboratory for Analytical Science of Food Safety and Biology Ministry of Education, College of Chemistry, Fuzhou University Fuzhou P. R. China

**Keywords:** carbon nitride, CO_2_ reduction, graphdiyne, photothermal catalysis, selective oxidation

## Abstract

Coupling photocatalytic CO_2_ reduction with organic oxidation promises enhanced solar energy conversion and atom economy but remains challenging due to the difficulty in orchestrating selective redox transformations while suppressing side reactions. Here, we report a metal‐free graphdiyne (GDY)/polymeric carbon nitride (PCN) heterojunction that achieves exceptional bifunctional performance in CO_2_ reduction coupled with tetrahydrofuran oxidation to *γ*‐butyrolactone. The optimized composite delivers a CO production rate of 55 µmol·h^−1^·g^−1^ with 95% selectivity, and a *γ*‐butyrolactone yield of 54% with near‐unity selectivity (> 99%) under mild photothermal conditions, representing 2.9‐fold and 6.8‐fold enhancements over thermally treated PCN, respectively. Mechanistic investigations reveal that GDY serves as a hole‐transport layer, generating a built‐in electric field that drives spatial separation of charge carriers. This configuration confines electrons on PCN for CO_2_ reduction while directing holes to GDY for tetrahydrofuran activation. Moreover, the metal‐free heterojunction suppresses over‐oxidation pathways that plague metal‐loaded systems, enabling remarkable selectivity control. This work establishes the GDY/PCN heterojunction as a powerful platform for cooperative photoredox catalysis and provides a blueprint for designing metal‐free heterojunctions toward sustainable synthesis.

## Graphical abstract

A fully metal‐free heterojunction constructed from graphdiyne (GDY) and polymeric carbon nitride (PCN) enables efficient coupling of CO_2_ reduction and tetrahydrofuran oxidation. GDY serves as a hole‐transport channel, establishing a built‐in electric field that drives spatial charge separation. The optimized catalyst achieves near‐unity selectivity for both CO (95%) and *γ*‐butyrolactone (> 99%), outperforming noble‐metal‐loaded systems.

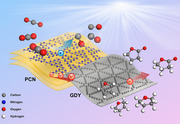

## 1 Introduction

**SCHEME 1:**
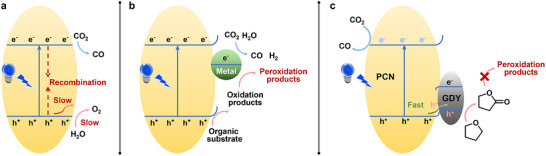
(a) Schematic diagram of solar‐driven CO_2_ reduction coupled with water oxidation. (b) Schematic diagram of metal‐catalyzed CO_2_ reduction coupled with organic oxidation. (c) Schematic diagram of the metal‐free heterojunction constructed from GDY and PCN for catalyzing CO_2_ reduction coupled with THF oxidation to synthesize GBL.

Solar‐driven CO_2_ reduction represents a pivotal strategy for achieving carbon neutrality [1]. Yet its efficiency is intrinsically capped by the high thermodynamic barrier and sluggish kinetics of the oxygen evolution reaction (OER) (Scheme 1a) [2, 3]. Coupling CO_2_ fixation with the selective oxygenation of organic substrates offers a compelling alternative that simultaneously bypasses the kinetic bottleneck of water oxidation and upgrades low‐value feedstocks into high‐value chemicals [4, 5]. However, orchestrating such dual‐functional catalysis presents a formidable challenge: it requires the precise engineering of band structures to thermodynamically straddle both redox potentials [6, 7], alongside rigorous control over interfacial charge dynamics to drive spatially distinct half‐reactions while suppressing bulk recombination [8, 9]. Meeting these requirements is essential for driving highly efficient and selective photoredox transformations. Without stringent selectivity control, the resulting products may hold lower value than the starting substrates, rendering the entire CO_2_ conversion process economically unattractive and undermining the very premise of resource upgrading [10, 11, 12, 13]. Despite its critical importance, the rational design of photocatalytic systems capable of achieving such precise selectivity remains largely unexplored.

Among potential oxidative half‐reactions, the direct transformation of cyclic ethers, such as tetrahydrofuran (THF), to ***γ***‐lactones (e.g., ***γ***‐butyrolactone, GBL) via sp^3^ C─H activation is especially appealing owing to the pharmaceutical and industrial importance of the lactone motif (Scheme S1) [14, 15, 16]. GBL serves as a high‐value intermediate in the production of pyrrolidones (e.g., N‐methyl‐2‐pyrrolidone), herbicides, and lithium‐ion battery electrolytes (Scheme S2) [17, 18, 19, 20]. However, this transformation embodies a classic selectivity paradox: the substrate α─C─H bonds are kinetically inert (bond dissociation energy: 92.0 kcal mol^−1^) [21], requiring a strong oxidative driving force, whereas the lactone product is thermodynamically prone to overoxidation or ring‐opening [22, 23]. In addition, thermochemical calculations indicate that synthesis of GBL and CO from THF and CO_2_ is endothermic (∆_r_H^0^ = +103.18 kJ·mol^−1^). As such, the use of photocatalysis to drive the outlined chemical reaction is fully justified by the thermodynamics. Existing industrial protocols rely on harsh conditions (high temperature/pressure) or stoichiometric toxic oxidants (e.g., Cr^VI^ and Mn^VII^) [24, 25, 26, 27], while current photocatalytic systems using metal‐based semiconductors (e.g., TiO_2_ and WO_3_) often suffer from poor selectivity (< 80%) [21, 28]. The potent oxidative holes or nonselective reactive oxygen species (ROS) generated by these metals frequently trigger ring‐opening or complete mineralization rather than the desired dehydrogenation (Scheme 1b) [29, 30]. Consequently, developing a mild, metal‐free catalytic system capable of coupling CO_2_ reduction with highly selective THF oxidation constitutes a grand challenge.

Polymeric carbon nitride (PCN) has emerged as a promising candidate for such dual‐functional catalysis due to its tunable band structure and chemical stability [31, 32, 33, 34]. Yet, the catalytic efficiency of pristine PCN is severely limited by sluggish charge‐carrier mobility and rapid bulk recombination [35, 36, 37]. While loading noble metal cocatalysts (e.g., Pt and Pd) can enhance charge separation, they introduce two interrelated selectivity issues [38, 39]. On the reduction side, these metals serve as efficient active sites for proton reduction, steering photogenerated electrons toward hydrogen evolution rather than the desired CO_2_ conversion [40]. On the oxidation side, they often catalyze the formation of nonselective ROS, which drive the deep oxidation of organic substrates via overoxidation and ring‐opening pathways rather than targeted dehydrogenation to valuable intermediates. Therefore, the development of a metal‐free heterojunction capable of accelerating charge transport while exerting precise control over reaction pathways represents a critical, yet unmet, challenge in the field.

To circumvent the inherent limitations of metal‐based cocatalysts, we construct a fully metal‐free graphdiyne/carbon nitride heterojunction designed to decouple the activity‐selectivity trade‐off in dual‐functional photocatalysis. Graphdiyne (GDY), a two‐dimensional carbon allotrope featuring sp‐ and sp^2^‐hybridized networks [41, 42, 43], possesses exceptional hole mobility (10^4^–10^5^ cm^2^·V^−1^·s^−1^) and a valence band structure ideally aligned with carbon nitride [44, 45, 46]. We postulate that the GDY interface serves a dual function: it acts as a high‐mobility conduit for rapid hole extraction, mitigating bulk recombination, while its unique acetylenic framework facilitates the specific adsorption and activation of tetrahydrofuran, directing oxidative equivalents toward selective C─H bond functionalization without compromising the lactone product. Mechanistic studies combining Kelvin probe force microscopy and density functional theory reveal that the work function difference between the two components generates a robust internal electric field at the interface. This built‐in field acts as a thermodynamic filter, spatially directing photogenerated electrons to the PCN surface for CO_2_ reduction while ballistically injecting holes into the high‐mobility GDY network for targeted THF oxidation (Scheme 1c). The resulting spatial isolation of redox centers, together with the intrinsically mild adsorption properties of the carbon framework, effectively suppresses both proton reduction and overoxidation side reactions. For the coupled conversion of CO_2_ and tetrahydrofuran to CO and *γ*‐butyrolactone, the optimized heterojunction 0.5G‐P delivers high selectivity, with 95% for CO and near‐unity (> 99%) for the GBL, while achieving a 6.8‐fold enhancement in GBL yield relative to thermally treated PCN. This work not only establishes GDY as a versatile platform for charge management in polymer photocatalysts but also provides a generalizable blueprint for designing selective, atom‐economical interfaces for cooperative photoredox transformations.

## 2 Results and Discussion

### 2.1 Structural Characterization of the Metal‐Free Heterojunction

**FIGURE 1:**
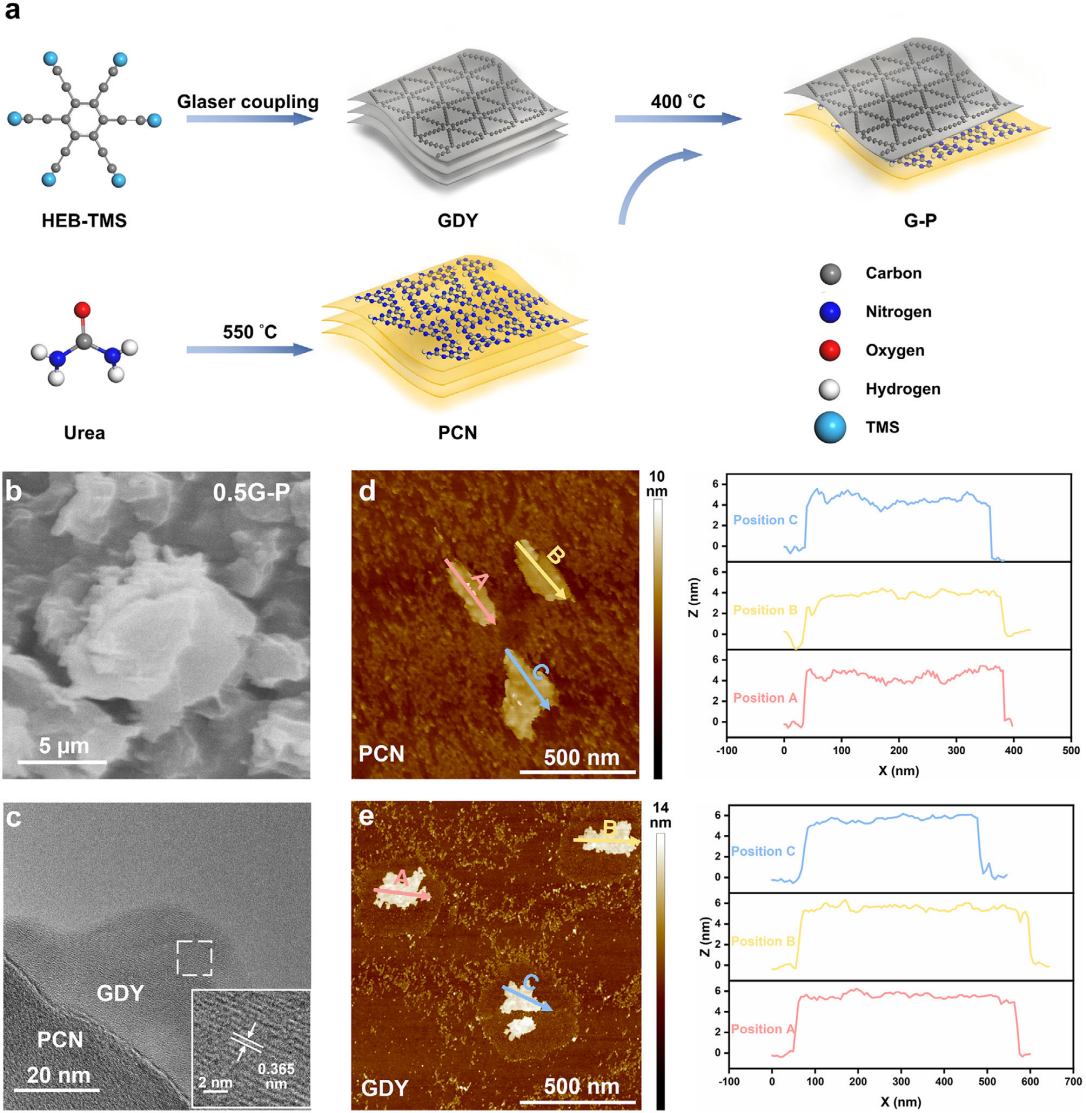
(a) Schematic preparation of G‐P composites photocatalyst. (b) SEM image of 0.5G‐P. (c) HR‐TEM image of 0.5G‐P. AFM images and corresponding thickness measurements of (d) the PCN, and (e) GDY nanosheets.

To engineer a robust, metal‐free heterojunction capable of efficient charge rectification, we employed a sequential electrostatic self‐assembly strategy followed by thermal consolidation. GDY and PCN were first synthesized via established protocols and subsequently integrated via electrostatic adsorption, ensuring intimate contact between the two distinct carbon allotropes prior to secondary calcination (Figure 1a). A series of composites with varying GDY loadings was prepared and evaluated, and the 0.5 wt% sample (0.5G‐P) was selected as a representative heterojunction for detailed characterization and comparison. For rigorous comparison, pristine PCN subjected to the identical thermal treatment was prepared as a control (denoted as PCN(400)). The morphological integrity and interfacial architecture of the synthesized catalysts were first examined by electron microscopy. Scanning electron microscopy (SEM) images reveal that the 0.5G‐P composite retains the characteristic laminar stacking morphology of pristine PCN, indicating that the incorporation of GDY does not disrupt the parent framework (Figures 1b and S1). Subsequently, high‐resolution transmission electron microscopy (HR‐TEM) was employed to directly interrogate the heterointerface (Figure 1c). A distinct phase boundary is observable, juxtaposing the amorphous texture of the PCN against the highly ordered, crystalline domain of GDY. Crucially, well‐defined lattice fringes with an interplanar spacing of 0.365 nm were clearly resolved, unambiguously assignable to the (002) plane of GDY [47]. This direct visualization of lattice fringes within the composite matrix provides compelling evidence for the formation of an intimate, coherent heterojunction, rather than a mere physical mixture. Zeta potential analysis provided further evidence for the self‐assembly of the composite material through electrostatic interactions (Figure S2).

To further validate the two‐dimensional nature of this assembly, atomic force microscopy (AFM) was utilized to probe the topographic height profiles of the constituent nanosheets (Figure 1d,e). Quantitative analysis of topographic height profiles across multiple regions confirms that both components exhibit uniform, ultrathin nanosheet morphologies, with PCN thicknesses of approximately 4–5 nm and GDY layers measuring 5–6 nm. These values are fully consistent with the laminar features observed by SEM, further supporting the formation of a 2D/2D van der Waals heterojunction. This face‐to‐face stacking geometry is particularly significant, as the extended interfacial contact area is expected to maximize electronic coupling and facilitate efficient charge transfer across the van der Waals gap. Moreover, textural analysis reveals that the 0.5G‐P composite retains a porous architecture similar to that of PCN, with Brunauer–Emmett–Teller (BET) surface area and pore size distribution essentially unchanged (Figure S3 and S4). Thermogravimetric analysis further demonstrates that the composite exhibits thermal stability comparable to that of pristine PCN (Figure S5). Collectively, these results demonstrate that the construction of the metal‐free heterojunction does not compromise the fundamental structural integrity or thermal stability of the catalyst.

**FIGURE 2:**
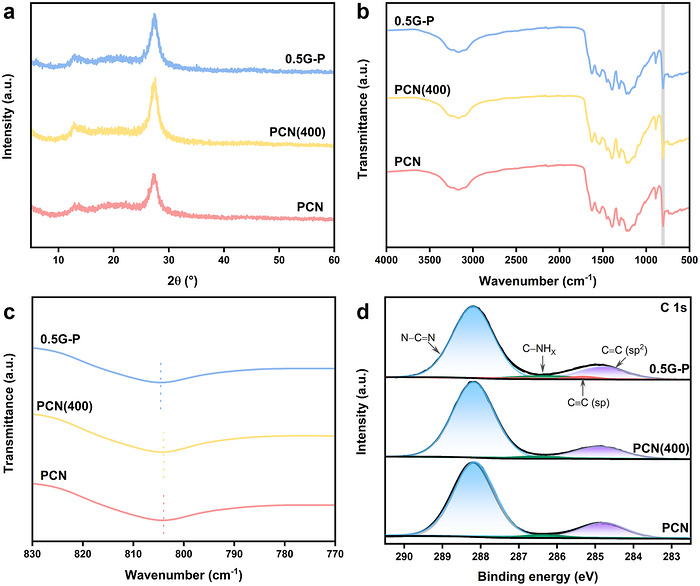
(a) XRD patterns of the PCN, PCN(400), and 0.5G‐P. (b) FT‐IR spectra of prepared samples. (c) Enlarged FT‐IR spectra (770–830 cm^−1^). (d) C 1s XPS of the samples.

The crystallographic fidelity of the host matrix is a prerequisite for stable photocatalysis. As evidenced by the x‐ray diffraction (XRD) patterns in Figure 2a, neither the secondary thermal treatment nor the incorporation of GDY compromises the bulk structure of the PCN. The composite retains the signature diffraction motifs of the parent material, specifically the in‐plane packing peak at approximately 13° (indexed to the (100) plane) and the dominant interlayer stacking reflection at ∼27° ((002) plane) [48, 49]. The preservation of these features confirms that the GDY nanosheets are successfully integrated into the PCN matrix without inducing collapse or distortion of the heptazine‐based framework. While the bulk crystal structure of PCN remains essentially unchanged upon composite formation, Fourier‐transform infrared (FT‐IR) spectroscopy reveals notable modifications to the local electronic environment. As shown in Figure 2b, both PCN samples and the 0.5G‐P composite exhibit the characteristic breathing vibration of the heptazine ring at 805 cm^−1^ [50, 51]. Notably, this vibrational mode undergoes a slight blueshift in the 0.5G‐P composite relative to pristine PCN (Figure 2c). This spectral shift cannot be attributed to simple physical mixing, but rather signifies enhanced *π*‐electron delocalization and intensified *π*–*π* stacking interactions at the heterointerface. These results provide compelling evidence that the GDY–PCN interface is governed by strong electronic coupling rather than mere physical contact, establishing an effective conductive channel that facilitates charge carrier migration across the heterojunction. Owing to the relatively low loading of GDY, the Raman spectrum of 0.5G‐P closely resembles that of PCN (Figure S6).

To unambiguously identify the chemical states at this interface, we employed x‐ray photoelectron spectroscopy (XPS). Survey spectra confirm the elemental purity of the system, showing C and N with trace surface oxygen (Figure S7a). As shown in Figure S7b, the N 1s XPS spectra indicate that the nitrogen coordination environment of PCN remains intact upon heterojunction formation. The C 1s spectra offer deeper insight into the bonding architecture. In Figure 2d, the PCN and PCN(400) controls exhibit the standard deconvoluted peaks: the dominant N─C═N coordination of the heptazine ring (∼288.2 eV), defect‐associated C─NH_X_ species (∼286.1 eV), and adventitious or graphitic sp^2^ carbon (∼284.8 eV) [52, 53, 54]. Crucially, the 0.5G‐P composite spectrum reveals the emergence of a unique component at 285.3 eV. This peak is the specific fingerprint of sp‐hybridized C≡C bonds, confirming the successful incorporation of the acetylenic linkages intrinsic to the GDY lattice [55].

### 2.2 Photocatalytic Performance Evaluation

**FIGURE 3:**
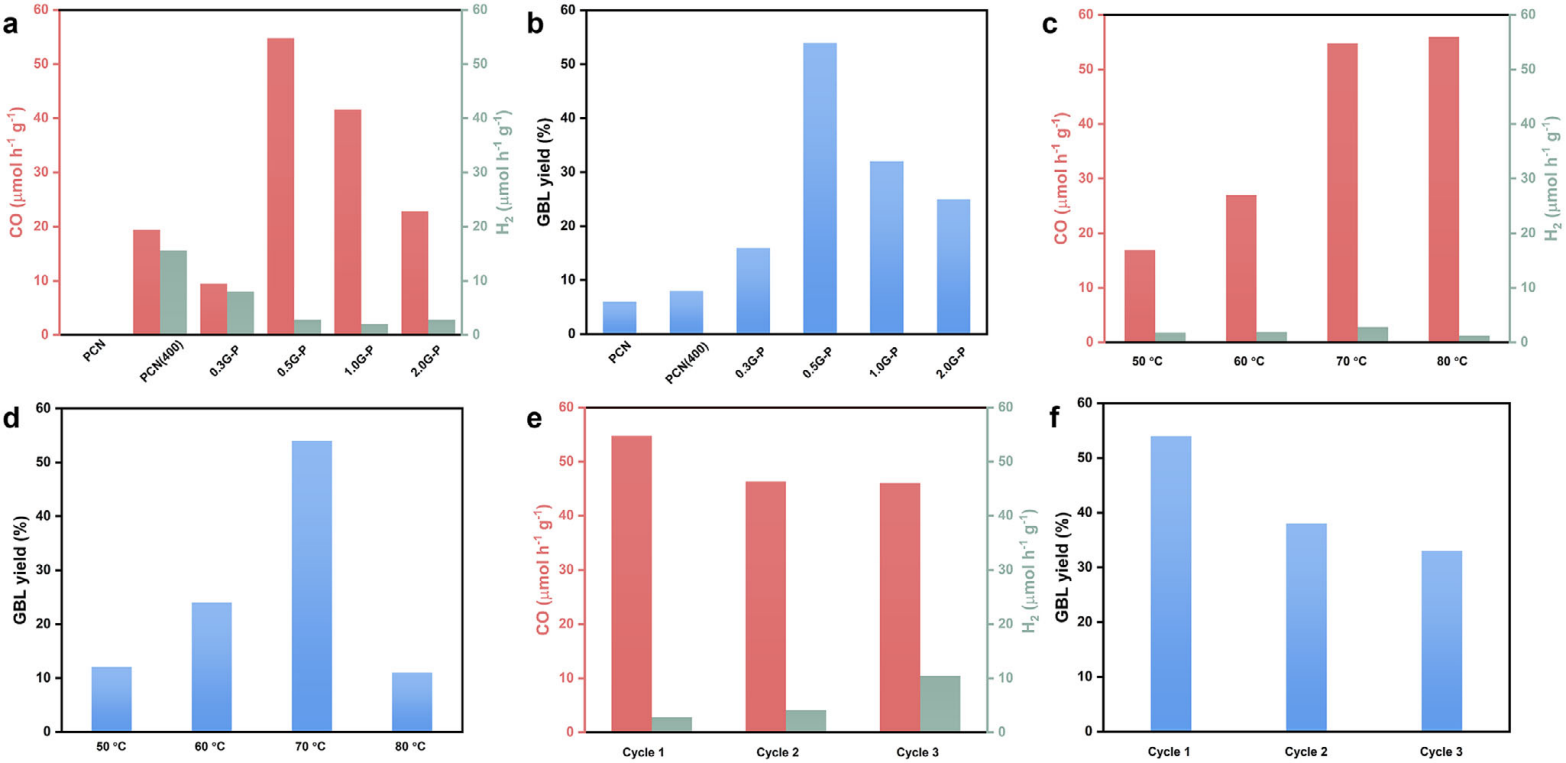
(a) CO and H_2_ production rate over different photocatalysts. (b) GBL yield over different photocatalysts. (c) CO and H_2_ production rate under different temperatures. (d) GBL yield under different temperatures. The cycle reaction of (e) CO and H_2_ production rate, and (f) GBL yield.

To demonstrate the practical utility of the engineered interface, we evaluated the catalysts in a dual‐functional redox system that simultaneously drives the reduction of CO_2_ to CO and the oxygenation of THF to GBL. As illustrated in Figure 3a, when pristine PCN was employed as the photocatalyst, only trace amounts of hydrogen were detected as the gaseous product, with no observable CO evolution. On the oxidation side, PCN afforded a GBL yield of 6% (Figure 3b). While the thermally treated control sample (PCN(400)) exhibited detectable CO_2_ reduction activity, it suffered from severe selectivity issues due to competitive proton reduction leading to hydrogen evolution, and its GBL yield remained modest at 8%. In stark contrast, the construction of the GDY/PCN heterojunction unlocked exceptional catalytic activity. The performance followed a distinct volcano‐type dependence on GDY loading, culminating in the optimized 0.5G‐P catalyst (0.5 wt% GDY). This metal‐free heterojunction achieved a maximal CO production rate of 55 µmol·h^−1^·g^−1^ with a remarkable selectivity of 95%—values that represent 2.6‐fold and 1.7‐fold enhancements, respectively, over the PCN(400) baseline. Notably, the CO yield and selectivity achieved by 0.5G‐P surpass that of most previously reported metal‐free carbon nitride‐based photocatalysts under comparable conditions, underscoring the superiority of the heterojunction design (Table S1). Simultaneously, on the oxidative terminus, 0.5G‐P delivered a GBL yield of 54% with > 99% selectivity, surpassing the control sample by a factor of 6.8 (Figure S8). The decline in activity at higher GDY loadings is attributed to an optical shielding effect: excessive black GDY competes with the photoactive PCN for incident photons, thereby dampening overall quantum efficiency. Control experiments were performed to validate the essential role of the heterojunction interface. A simple physical mixture of GDY and PCN exhibited substantially lower photocatalytic activity than the 0.5G‐P composite, confirming that thermal treatment is necessary to construct an effective heterojunction interface (Figure S9). Furthermore, the possibility of activity enhancement from trace copper impurities was excluded by control experiments showing that copper doping did not improve the catalytic performance (Figure S10). Notably, heterojunctions constructed from GDY and PCN derived from different precursors all exhibited enhanced activity, underscoring the universal role of GDY in boosting PCN performance (Figure S11). We further probed the kinetics of the system by modulating the reaction temperature (Figure 3c,d). Below 70°C, increased thermal energy favorably promoted charge‐carrier separation and surface kinetics, enhancing both CO generation and GBL yield. However, the system exhibited a sharp drop in efficiency once the temperature exceeded the boiling point of THF, likely due to phase instability and substrate volatilization. Increasing the catalyst dosage from 5 to 10 mg led to a substantial improvement in activity, whereas a further increase to 20 mg produced only a negligible additional enhancement (Figure S12). Furthermore, the heterojunction exhibits the highest photocatalytic activity under 420 nm LED illumination (Figure S13).

To assess the merit of the 0.5G‐P heterojunction, comparative experiments were conducted using PCN modified with conventional noble metal cocatalysts (Cu, Co, Pt) via photo‐deposition. While noble metals are widely regarded as efficient charge collectors, the metal‐free 0.5G‐P heterojunction unexpectedly delivered superior CO production rates and selectivity (Table S2). More notably, in the oxidation half‐reaction, metal‐loaded catalysts promoted nonselective over‐oxidation pathways, resulting in substantially lower GBL yields compared to the metal‐free system. This underscores the unique advantage of the GDY/PCN interface: it provides a precise electronic environment that facilitates targeted redox transformations without the thermodynamic sink often introduced by metal nanoparticles. The photocatalytic nature of the observed activity was rigorously validated through control experiments, which confirmed that no products were generated in the absence of either light or catalyst, establishing a photon‐driven mechanism (Table S3). Isotopic labeling with ^13^CO_2_ provided unambiguous evidence that the evolved CO originates exclusively from the gaseous feedstock (Figure S14). In addition, isotopic labeling with H_2_^18^O further confirmed that the oxygen atom incorporated into the resulting GBL originated from CO_2_. Furthermore, recycling tests revealed that while the 0.5G‐P composite underwent a slight decrease in activity over three consecutive cycles, its exceptional product selectivity remained fully preserved, demonstrating the robustness of the heterojunction for sustained photocatalytic operation (Figure 3e,f). Weighing the catalyst after each photocatalytic cycle reveals that the progressive decay in performance is primarily attributable to physical loss of the catalyst during recovery (Table S4).

### 2.3 Interfacial Kinetics and Mechanism Study

**FIGURE 4:**
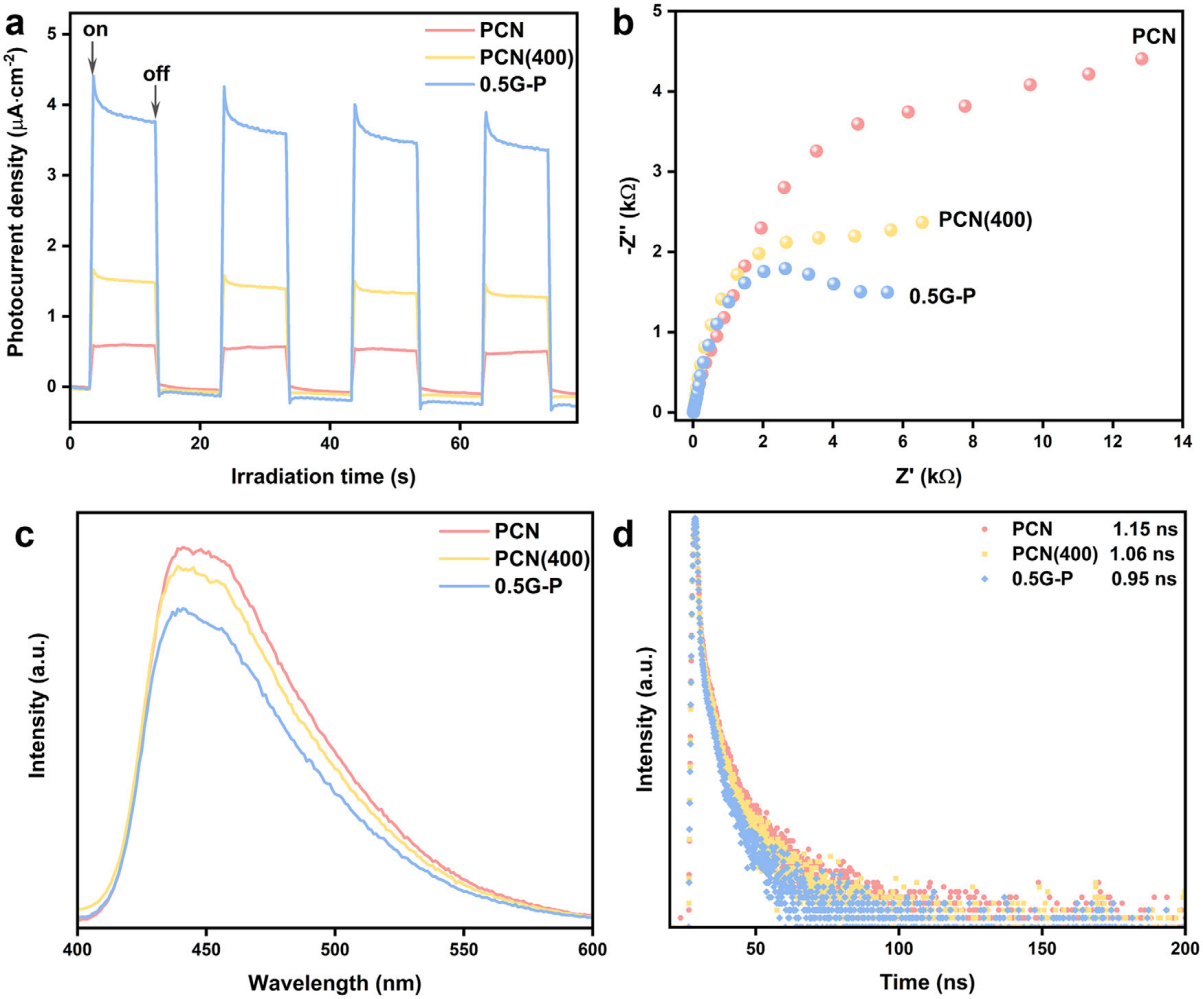
(a) Photocurrent response, (b) EIS plots, (c) steady‐state PL spectra, and (d) time‐resolved PL spectra of the samples.

To elucidate the mechanistic origin of the enhanced catalytic performance, a systematic investigation of charge‐carrier dynamics within the heterojunction was undertaken. Ultraviolet–visible diffuse reflectance spectroscopy (UV–vis DRS) reveals that the optical absorption edge of the 0.5G‐P composite remains nearly identical to that of pristine PCN, indicating that the incorporation of a low loading of GDY does not alter the intrinsic bandgap of the host material (Figure S15). This observation implies that the marked improvement in photocatalytic activity does not arise from extended light harvesting, but rather from a fundamental modulation of the charge separation kinetics. The steady‐state photocurrent response shows that the 0.5G‐P composite affords a photocurrent density of 3.9 µA·cm^−2^, approximately eight times that of pristine PCN (0.5 µA·cm^−2^) (Figures 4a and S16). Such a pronounced increase in photocurrent reflects a substantial suppression of bulk charge recombination, enabling more efficient extraction of photogenerated carriers to the electrode surface [56]. Electrochemical impedance spectroscopy further corroborates this interpretation (Figures 4b and S17). The Nyquist plot of the 0.5G‐P electrode shows a markedly reduced arc radius compared to that of pristine PCN, indicating a lower interfacial charge‐transfer resistance. Collectively, these results demonstrate that the construction of the GDY/PCN heterojunction significantly facilitates interfacial charge migration and suppresses charge recombination, thereby accounting for the observed enhancement in photocatalytic performance.

The photophysics of the excited states were further interrogated using steady‐state and time‐resolved photoluminescence (PL) spectroscopy. As shown in Figure 4c, the steady‐state PL spectra reveal a pronounced quenching of the intrinsic fluorescence emission for the 0.5G‐P composite relative to pristine PCN. This quenching behavior suggests that the formation of the heterojunction facilitates charge transfer between the two components, potentially suppressing radiative recombination pathways. Quantitative insight into this process is provided by time‐resolved PL decay profiles (Figure 4d). The average fluorescence lifetime decreases from 1.15 ns for PCN to 0.95 ns for the 0.5G‐P composite. In this heterostructure, the accelerated decay kinetics do not arise from defect‐induced trapping but rather reflect rapid nonradiative charge transfer from PCN to GDY. This assignment is consistent with GDY's role as an effective charge‐carrier acceptor, in which the interfacial charge‐transfer process kinetically outcompetes intrinsic recombination, thereby prolonging the availability of charge carriers for subsequent surface redox reactions. These photophysical observations are fully consistent with the photoelectrochemical data and collectively confirm that the GDY/PCN heterojunction facilitates spatial charge separation and directed carrier migration.

**FIGURE 5:**
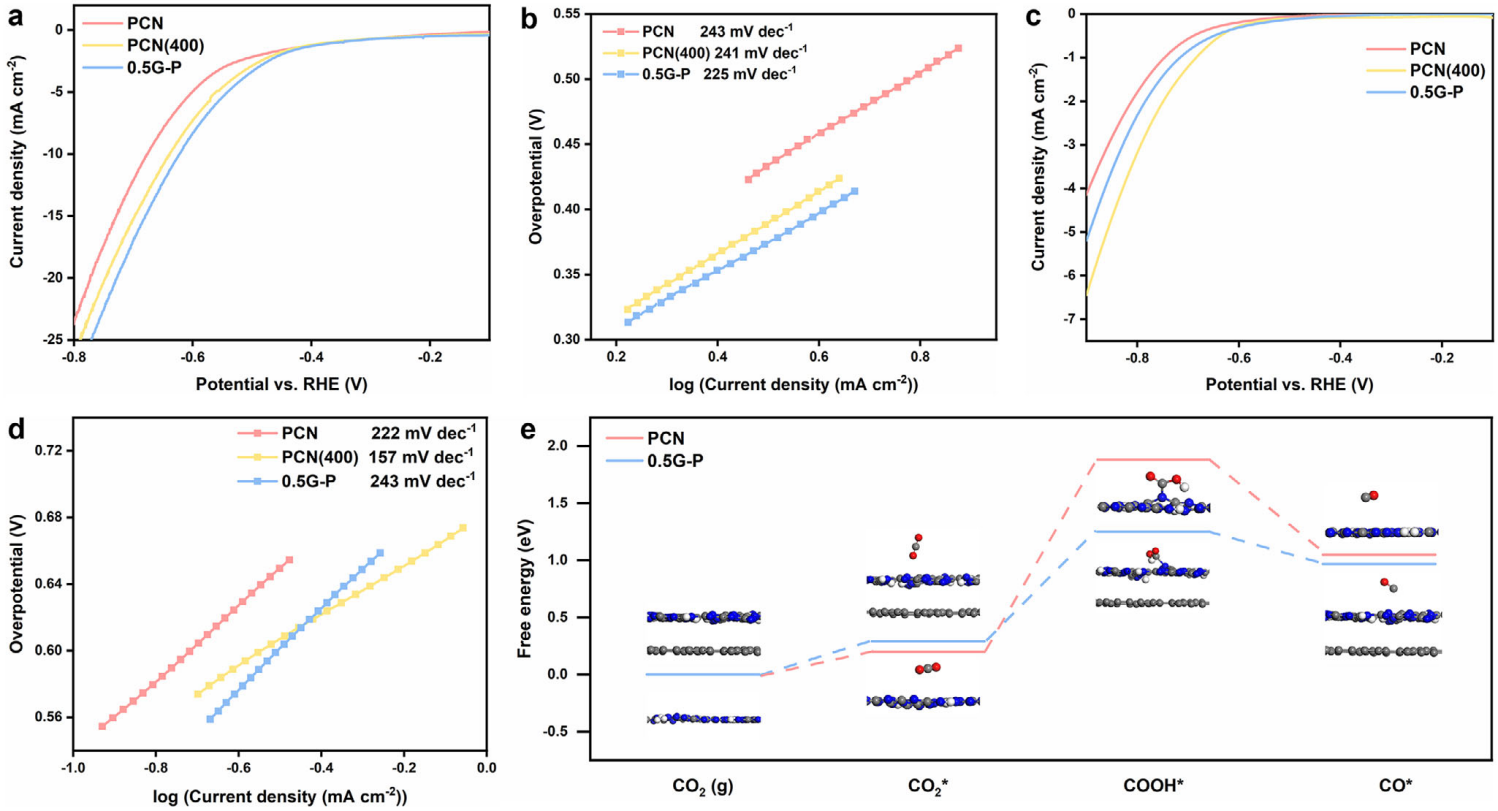
(a) LSV curves, and (b) Tafel plots of the samples tested in a CO_2_‐saturated electrolyte. (c) LSV curves, and (d) Tafel plots of the samples tested in a N_2_‐saturated electrolyte. (e) Full free energy for CO_2_ reduction on PCN and G‐P photocatalysts by DFT calculations. Atoms in gray, blue, red, and white represent C, N, O, and H, respectively.

Having established via Mott–Schottky analysis that the band edge positions are thermodynamically sufficient for the target redox reactions, we turned our attention to the kinetic factors governing the enhanced activity and, crucially, the selectivity (Figure S18). Contact angle measurements demonstrated that the 0.5G‐P composite exhibited the best dispersibility in aqueous solvents (Figure S19). Linear sweep voltammetry (LSV) under CO_2_ saturation reveals that the 0.5G‐P composite and PCN(400) exhibit a marked anodic shift in onset potential alongside a higher current density compared to PCN (Figure 5a). Specifically, the cathodic onset potential of 0.5G‐P is substantially more positive than that of both PCN and PCN(400), accompanied by a significantly higher current density across the entire potential window. These observations indicate that thermal treatment alone reduces the activation energy for CO_2_ reduction to some extent, while the formation of the GDY/PCN heterojunction further lowers this kinetic barrier. Additional kinetic insights were obtained from Tafel analysis (Figure 5b). Among all samples, the 0.5G‐P composite exhibits the smallest Tafel slope, signifying that the rate‐limiting electron transfer from the catalyst surface to adsorbed CO_2_ species is markedly facilitated at the heterojunction interface. This enhanced substrate affinity is further corroborated by CO_2_ temperature‐programmed desorption profiles, which confirm the presence of stronger CO_2_ adsorption sites on the 0.5G‐P composite relative to the controls (Figure S20).

In aqueous photocatalysis, the hydrogen evolution reaction (HER) represents a competing pathway that often undermines the selectivity of CO_2_ reduction, as protons can readily capture photogenerated electrons at the catalyst surface. To assess the relative propensity for HER in the prepared samples, LSV measurements were recorded under N_2_ atmosphere (Figure 5c,d). Among all the samples, PCN(400) exhibits the highest cathodic current density across the entire potential window, indicating that the thermally treated carbon nitride surface facilitates facile proton reduction, thereby promoting hydrogen evolution. This observation is consistent with the photocatalytic results, wherein PCN(400) produced a substantial amount of H_2_ alongside CO, accounting for its compromised CO selectivity. In contrast, the 0.5G‐P composite exhibits a markedly lower cathodic current density, indicating a higher HER overpotential. This kinetic penalty for proton reduction, combined with the enhanced CO_2_ activation kinetics, creates a favorable kinetic divergence: the heterojunction interface synergistically lowers the barrier for CO_2_ reduction while simultaneously impeding the competing HER pathway. As a result, photogenerated electrons are preferentially channeled toward carbon fixation, accounting for the superior CO selectivity observed with the 0.5G‐P system.

Density functional theory calculations were performed to provide an atomistic rationale for the observed kinetic preference. As shown in Figures 5e and S21–S24, the carbon dioxide molecules preferentially adsorb on the PCN surface rather than on GDY, establishing PCN as the primary active site for substrate activation. The computed free‐energy landscape identifies the protonation of adsorbed CO_2_ to form the *COOH intermediate as the rate‐determining step in CO_2_ reduction. On the pristine PCN surface, this step encounters a substantial energy barrier of 1.68 eV. Remarkably, at the GDY‐PCN heterojunction interface, this barrier is significantly reduced to 0.96 eV, indicating that the construction of the heterojunction markedly facilitates this critical activation step. Furthermore, the composite surface exhibits a lower desorption energy for the CO product (−0.97 eV), which promotes rapid product release and catalyst regeneration, thereby mitigating potential site poisoning. Collectively, these theoretical findings, in conjunction with the experimental results, demonstrate that the GDY‐modified interface fundamentally alters the reaction landscape, enabling efficient, selective, and sustained CO_2_ conversion.

**FIGURE 6:**
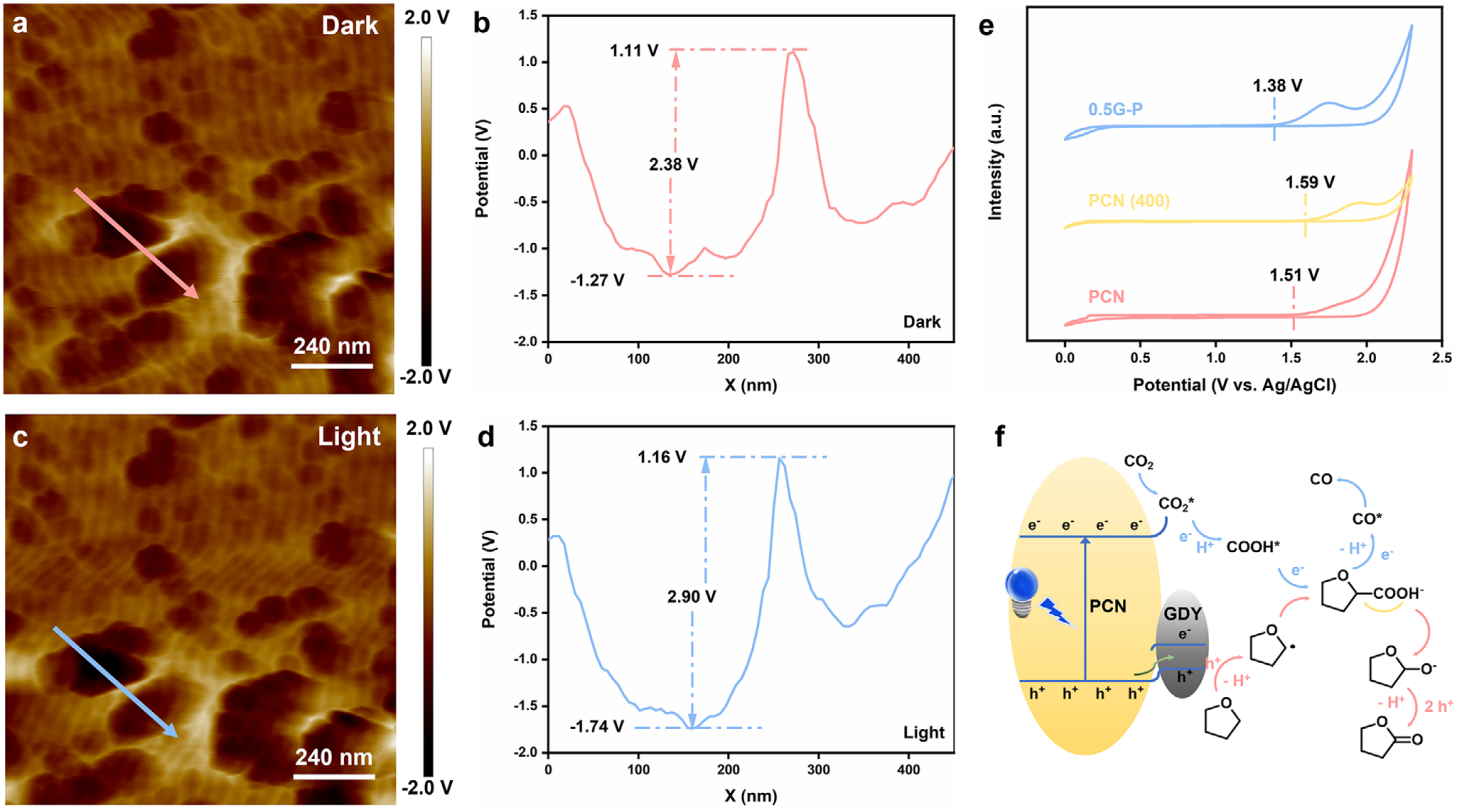
Image (a) and distribution (b) of potential difference at the interface of G‐P in a dark environment. Image (c) and distribution (d) of potential difference at the interface of G‐P under illumination. (e) CV curve of THF on PCN, PCN(400), and 0.5G‐P. (f) Schematic diagram of photocatalytic CO_2_ reduction integrated with THF oxidation.

To unambiguously resolve the directionality of photogenerated charge transfer within the heterostructure, a model interface was constructed by growing a uniform GDY film in situ on FTO glass [57], followed by the deposition of PCN nanosheets (Figures S25–S27). This planar architecture enabled spatial mapping of surface potential variations using Kelvin probe force microscopy. Under dark conditions (Figure 6a,b), a distinct contact potential difference is observed, with PCN domains exhibiting a lower surface potential relative to the GDY support. Upon illumination, the surface potential landscape undergoes a pronounced redistribution, directly reflecting the migration and accumulation of photogenerated charge carriers (Figure 6c,d). Specifically, the surface potential in the PCN region decreases markedly from −1.27 to −1.74 V. This negative shift indicates the accumulation of photogenerated electrons, confirming PCN as the reductive active site for CO_2_ conversion. Conversely, the GDY domain exhibits a positive potential shift from 1.11 to 1.16 V, characteristic of hole accumulation, validating GDY's role as an efficient hole acceptor.

This spatial separation of redox centers, with electrons confined to PCN and holes extracted to GDY, effectively suppresses charge recombination and sustains the long‐lived carriers essential for multi‐electron redox reactions. To verify that this hole extraction translates into enhanced oxidative kinetics, the electro‐oxidation of tetrahydrofuran was evaluated on the catalyst surfaces (Figure 6e). The 0.5G‐P interface exhibits the lowest onset potential (1.38 V vs. Ag/AgCl) and the highest current density among all samples, demonstrating that the photogenerated holes transported to the GDY frameworks are not merely trapped but remain energetically available for substrate activation. These results confirm that the GDY/PCN heterojunction significantly lowers the kinetic barrier for THF oxidation, enabling efficient and selective oxidative esterification. To further corroborate the function of GDY in tetrahydrofuran activation, electron paramagnetic resonance (EPR) spectroscopy coupled with spin‐trapping techniques was employed using 5,5‐dimethyl‐1‐pyrroline N‐oxide (DMPO) as the spin trap. Under photoirradiation, characteristic EPR signals corresponding to the DMPO‐trapped α‐oxyalkyl radical adduct were observed for the 0.5G‐P composite, whereas only negligible signals were detected for pristine PCN (Figures S28 and S29). This observation provides direct evidence that the GDY interface facilitates the photogenerated hole‐mediated C─H bond activation of THF to generate the key *α*‐oxyalkyl radical intermediate [58]. Furthermore, radical trapping experiments show that the addition of 2,2,6,6‐tetramethylpiperidin‐1‐oxyl (TEMPO) or butylated hydroxytoluene (BHT) significantly suppressed the yields of both CO and GBL, confirming that the *α*‐oxyalkyl radical is the key intermediate in the oxidation of THF to GBL (Figure S30).

Based on the above analyses, a mechanism for the G‐P heterojunction is proposed (Figure 6f). Upon formation of an intimate interface between semiconducting PCN and semimetal‐like GDY, equilibration of their Fermi levels occurs, driven by the work function difference between the two components. This charge redistribution generates a space‐charge region, with a depletion layer forming on the PCN side and an accumulation layer on the GDY side, thereby establishing a robust built‐in electric field directed from PCN toward GDY. Under visible light irradiation, this internal electric field serves as the primary driving force for spatial charge separation: photogenerated electrons remain confined within the conduction band of PCN to reduce adsorbed CO_2_* to the COOH* intermediate. While holes are rapidly extracted across the interface to the GDY surface, where they oxidize THF via dehydrogenation to form an *α*‐oxyalkyl radical intermediate. The COOH* intermediate is further reduced and then attacks the electrophilic *α*‐oxyalkyl radical, forming a coupling intermediate. Subsequent elimination of a CO molecule, followed by hole oxidation, affords the target product GBL. The exceptionally high hole mobility of the GDY network ensures that these oxidative equivalents are delivered to surface reaction sites more rapidly than the charge‐recombination timescale, enabling the simultaneous and efficient production of CO and γ‐butyrolactone with high selectivity.

## 3 Conclusion

In summary, we have constructed a fully metal‐free GDY/PCN heterojunction that successfully decouples the activity‐selectivity trade‐off inherent to dual‐functional photocatalysis. Mechanistic investigations reveal that GDY serves a dual role as both an efficient hole‐transport layer and a selective substrate activator. The work‐function difference between the two components generates a robust built‐in electric field at the heterointerface, which spatially directs photogenerated electrons to the PCN surface for CO_2_ reduction while rapidly extracting holes into the GDY network for targeted tetrahydrofuran activation. This spatial isolation of redox centers, combined with the intrinsically mild adsorption properties of the metal‐free framework, effectively suppresses both competitive proton reduction and overoxidation side reactions that typically compromise the selectivity of conventional metal‐loaded systems. The successful demonstration of this cooperative photoredox system establishes GDY‐based heterojunctions as a versatile platform for charge management in polymer photocatalysts and provides a generalizable strategy for designing selective, atom‐economical interfaces, paving the way toward sustainable photocatalytic technologies for simultaneous fuel production and chemical synthesis.

## Conflicts of Interest

The authors declare no conflicts of interest.

## Supporting information




**Supporting File**: The authors have cited additional references within the Supporting Information.

## Data Availability

The data that support the findings of this study are available from the corresponding author upon reasonable request.
