# Supplementary material for: Graphdiyne as a Hole‐Transport Channel in Carbon Nitride Heterojunctions for Synergistic CO2 Reduction and γ‐Butyrolactone Synthesis
Source: Angew Chem Int Ed Engl. 2026 May 17;65(28):e3147768. doi: 10.1002/anie.3147768 (PMC13340512; doi:10.1002/anie.3147768)
Supplement: Supplementary file 1 — Supporting File: The authors have cited additional references within the Supporting Information. [file ANIE-65-e3147768-s001.docx]

**Supplementary Materials**

**Graphdiyne as a Hole-Transport Channel in Carbon Nitride Heterojunctions for Synergistic CO_2_ Reduction and** **γ-Butyrolactone Synthesis**

Xuan Zhang^1^, Junqi Chai^1^, Chong Wang^2^*, Wei Lin^1^, Oleksandr Savateev^2^, Jimmy C. Yu^2^, and Jiajia Cheng^1^*

1. State Key Laboratory of Chemistry for NBC Hazards Protection, State Key Laboratory of Photocatalysis on Energy and Environment, Sino-UK International Joint Laboratory on Photocatalysis for Clean Energy and Advanced Chemicals & Materials, College of Chemistry, Fuzhou University, Fuzhou 350116, P. R. China. Email: [jjcheng@fzu.edu.cn](mailto:jjcheng@fzu.edu.cn)
2. Department of Chemistry, The Chinese University of Hong Kong, Shatin, 999077 Hong Kong SAR, P. R. China. Email: [chongwang@cuhk.edu.hk](mailto:chongwang@cuhk.edu.hk)

**S1. Experimental Procedures**

**Materials:** All reagents and solvents were used as received without further purification. Reagents and solvents were bought from Energy Chemical, Sinopharm, and Aladdin.

**Synthesis of polymeric carbon nitride (PCN):** Urea (10 g) was heated to 550 ℃ for 4 h at a rate of 2 ℃ min^–1^ in the air atmosphere. The product was washed with deionized water and then dried at 70 ℃ overnight. This sample is denoted as PCN.

**Synthesis of graphdiyne (GDY):** Hexakis[(trimethylsilyl)ethynyl]benzene was synthesized according to a previous method.^[1]^ In order to obtain the powder instead of film, we used CuI powder instead of copper foil. The black GDY powder was washed with 1 M HNO_3_ and deionized water to remove Cu, then drying at 70 ℃ overnight. The sample is denoted as GDY.

**Synthesis of xG-P:** In a typical process, x mg (x = 0, 0.6, 1, 2, 4) GDY was dispersed in 45 mL of methanol via sonication for 2 h. Separately, 200 mg of PCN was sonicated in 100 mL of deionized water for 2 h. Subsequently, the two suspensions were mixed and sonicated for an additional 1 h. After solvent removal, the resulting powder was heated at 400 °C for 2 h under a nitrogen atmosphere with a heating rate of 4 ℃ min^-1^. According to the different weight ratios of GDY, the samples are denoted as PCN(400), 0.3G-P, 0.5G-P, 1.0G-P and 2.0G-P.

**Synthesis of PCN modified with conventional noble metal cocatalysts:** Noble metal co-catalysts were loaded onto PCN via a photodeposition method.^[2]^ Typically, 100 mg of PCN was dispersed in 100 mL of deionized water containing 0.5 wt% metal precursors, specifically Cu(NO_3_)_2_·6H_2_O, CoCl_2_·6H_2_O, and H_2_PtCl_6_·6H_2_O, respectively. Subsequently, 10 mL of triethanolamine was added, and the atmosphere was replaced with nitrogen. The suspension was then irradiated under a 300 W Xenon lamp for 2 hours. The resulting precipitate was collected, washed with deionized water and ethanol, and dried at 70 ℃ overnight. The obtained products were denoted as Cu-PCN, Co-PCN, and Pt-PCN, respectively.

**S2. Characterizations**

Scanning electron microscope was performed on a field emission scanning electron microscope (SEM, SU-8010, HITACHI). High resolution transmission electron microscope (HRTEM) was performed on a transmission electron microscope (TEM, FEI, Talos). Powder X-ray diffraction (XRD) tests were conducted on a Rigaku Minflex 600 Advance X-ray instrument (Cu Kα1 radiation, k = 1.5406 Å) at a current of 40 mA and a voltage of 40 kV. X-ray photoelectron spectroscopy (XPS) data were obtained on the Thermo ESCALAB250 instrument with a monochromatized Al Kα line source (200 W). Fourier transform infrared (FT-IR) spectra were acquired on a Nicolet IS-50 instrument. Raman spectra were collected on Horiba LABRAM HR Evolution spectrophotometer. The Micromeritics 2460 adsorption analyzer was used for N_2_ (77 K) adsorption-desorption measurement and BET analysis. Raman spectra were collected on Horiba LABRAM HR Evolution spectrophotometer. The thermal stability of catalysts were determined by thermogravimetric analysis/differential scanning calorimetry (TGA/DSC; STARe System) from 20 to 1000 °C with a ramp rate of 10 °C min^-1^. The ultraviolet visible (UV-Vis) diffuse reflectance spectra (DRS) of the samples were conducted on Varian Cary 500 Scan UV-Vis spectrophotometer and PerkinElmer UV/VIS.NIR Spectrometer Lamdba 950. Electron paramagnetic resonance (EPR) measurements were carried out on a Bruker model A300 spectrometer. Steady-state photoluminescence (PL) spectra were carried out on Horiba Fluorolog TCSPC spectrophotometer using Xr lamp as excitation light source. The electrochemical impedance, Mott-Schottky, photocurrent response, and linear sweep voltammetry (LSV) of the catalysts were measured on the electrochemical workstation (Bio-Logic, VSP-300). The AFM image was obtained with a ScanAsyst mode on a Bruker Dimension ICON (probe: ScanAsyst-AIR) and Kelvin probe force microscopy (KPFM) surface potential distribution was recorded using an SCM-PIT probe in AFM. ^1^H NMR spectra are reported in parts per million (ppm) downfield relative to D_2_O (4.79 ppm) and all ^13^C NMR spectra are reported in ppm relative to D_2_O (4.79 ppm) unless stated otherwise.

**S3. Photo-electrochemical measurements**

Electrochemical measurements were conducted in a standard three-electrode system, using a Pt plate as the counter electrode and a silver chloride electrode (Ag/AgCl) as the reference electrode. The working electrode was prepared on a F-doped SnO_2_-coated glass (FTO glass), which was cleaned by sonication in ethanol for 30 min and dried at 25 ℃. The working electrodes were prepared by dip-coating as follows: 2 mg of the sample was dispersed in 2 mL of N,N-dimethylformamide (DMF) by sonication to give a slurry mixture. 10 μL of the slurry was spread onto the pretreated FTO glass. The electrolyte was a 0.2 M Na_2_SO_4_ aqueous solution without additive (pH = 7). LSV measurements for CO_2_ reduction were performed using an electrolyte of 0.1 M tetrabutylammonium hexafluorophosphate in acetonitrile. Electrochemical impedance spectroscopy and the data for Mott-Schottky analysis were acquired without irradiation. Photocurrent response experiments were conducted by alternating the periods of the electrode irradiation with light, followed by the current measurements in the dark.

**S4. Photocatalytic CO_2_ reduction and γ-butyrolactone synthesis test**

10 mg of the photocatalyst and 0.25 mmol of tetrahydrofuran (THF) were dispersed in 2 mL of deionized water within a sealed Schlenk tube. The atmosphere was exchanged for CO_2_ via three freeze-pump-thaw cycles. A 50 W LED lamp (420 nm) was used as the light source. The reactor was placed in an oil bath at 70 ℃ and the reaction was allowed to proceed for 24 hours. The gaseous products were analyzed using an Agilent 7890B gas chromatograph, while the liquid products were analyzed by ^1^H NMR spectroscopy. The photographic image of the reaction setup is presented below.


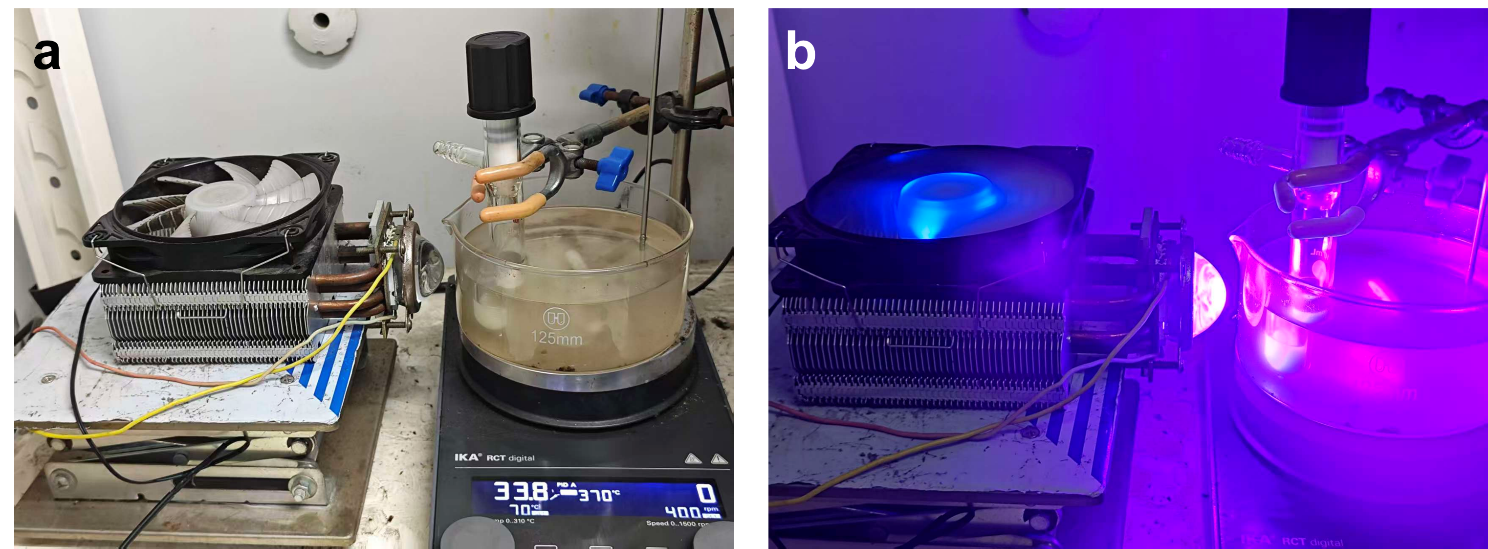


**S5. DFT calculations**

DFT calculations were conducted through the Vienna ab initio Simulation Package (VASP) with the projector augment wave method.^[3,4]^ Generalized gradient approximation of the Perdew-Burke-Ernzerhof (PBE) functional was used as the exchange-correlation functional.^[5]^ The Brillouin zone was sampled with gamma points for surface calculation. The cutoff energy was set as 500 eV, and structure relaxation was performed until the convergence criteria of energy and force reached 1 × 10^-5^ eV and 0.02 eV Å^-1^, respectively. A vacuum layer of 15 Å was constructed to eliminate interactions between periodic structures of surface models. The van der Waals (vdW) interaction was amended by the zero damping DFT-D3 method of Grimme.^[6]^

The adsorption energy (ΔE_ads._) of adsorbate adsorption on surface is defined as

ΔE_ads._ = E(*adsorbate) − E(*) − E(adsorbate)

where E(*adsorbate) and E(*) are the total energy of surface systems with and without adsorbate, respectively, E(adsorbate) is the energy of an isolated adsorbate. According to this definition, negative adsorption energy suggests that the adsorption process is exothermic and the adsorption system is thermodynamically stable. Contrarily, a positive value corresponds to an endothermic and unstable adsorption.

The Gibbs free energy was calculated as:

ΔG = ΔE + ΔE_ZPE_ −TΔS

where the ΔE, ΔE_ZPE_, and ΔS are electronic energy, zero-point energy, and entropy difference between products and reactants. The zero-point energies of isolated and absorbed intermediate products were calculated from the frequency analysis.^[7]^ The vibrational frequencies and entropies of molecules in the gas phase were obtained from the National Institute of Standards and Technology (NIST) database.^[8,9]^


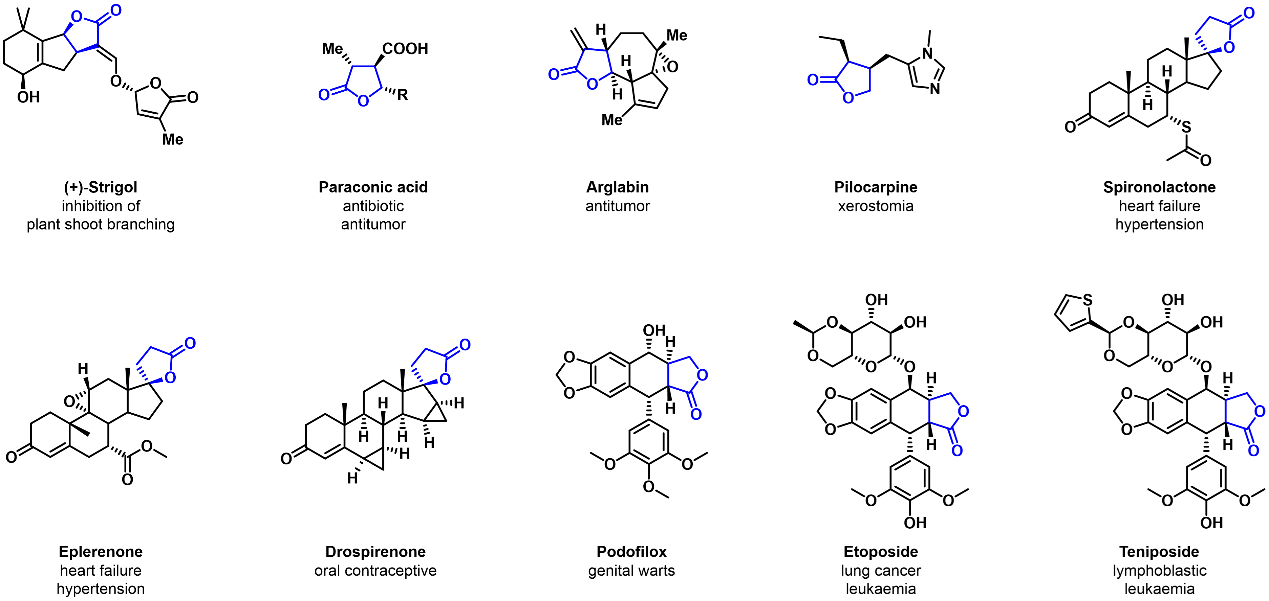


**Scheme S1.** Drug molecules which contain γ-butyrolactone core.

The core structural framework shared by numerous natural products built upon the γ-butyrolactone motif exhibits a broad spectrum of biological activities and is of particular significance for the development of physiological and therapeutic agents.^[10]^


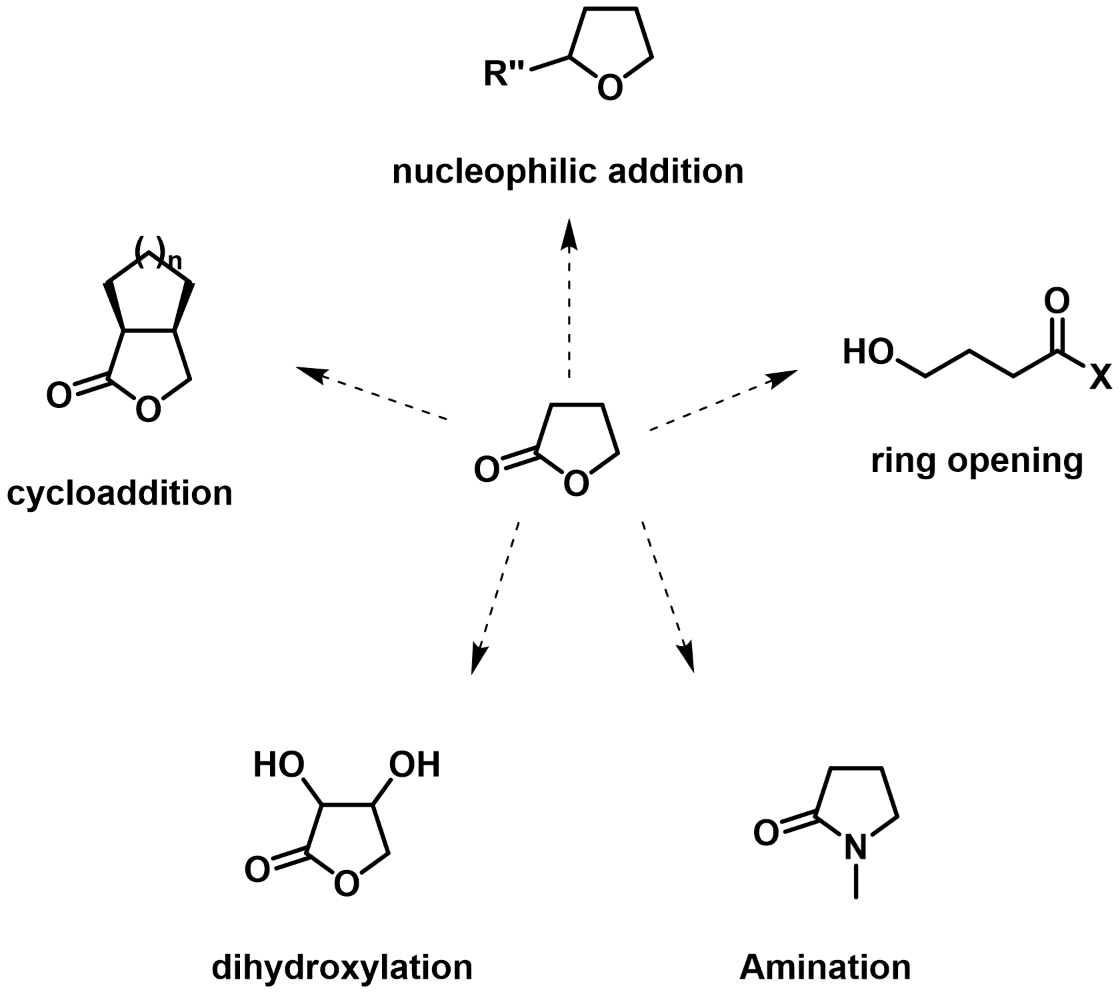


**Scheme S2.** Illustrative transformations of γ-butyrolactones. Optically active γ-butyrolactones represent a significant class of building blocks, widely employed in the synthesis of diverse biologically active compounds and complex molecules.^[11]^ These versatile intermediates can be elaborated into various important multifunctional building blocks through numerous transformations (including nucleophilic addition, cycloaddition, ring opening, dihydroxylation, and amination reactions).


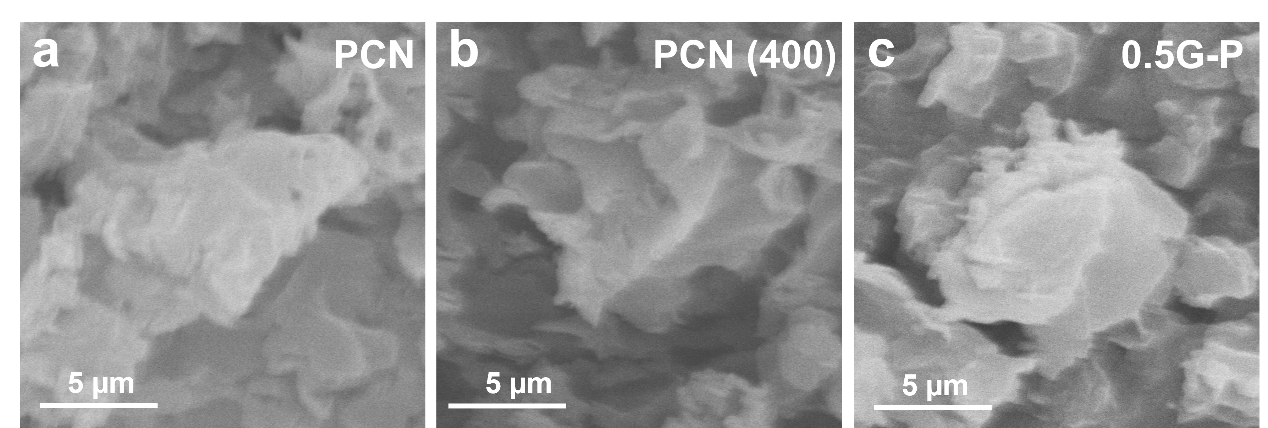


**Figure S1.** SEM images of (a) PCN, (b) PCN(400), and (c) 0.5G-P.

All the catalysts exhibited a morphology characterized by the stacking of nanosheets.


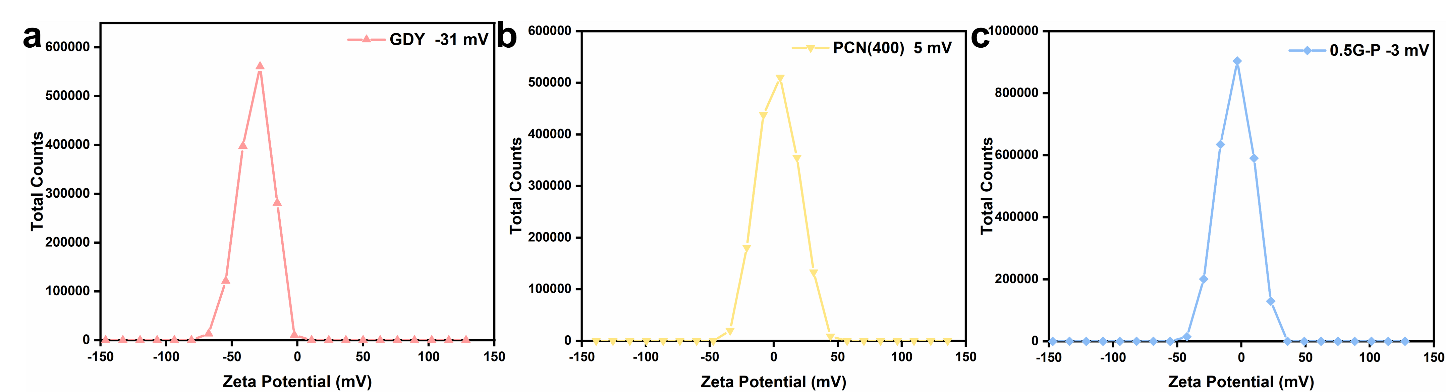


**Figure S2.** Zeta potential plots of the photocatalysts: (a) GDY, (b) PCN(400), (c) 0.5G-P.

The GDY surface exhibited a negative charge, while the surface of PCN(400) displayed a weak positive charge, indicating that the two materials could combine via electrostatic self-assembly. Furthermore, the surface potential of the resulting 0.5G-P composite fell between those of the two components, providing additional evidence for the successful synthesis of the catalyst through electrostatic interactions.


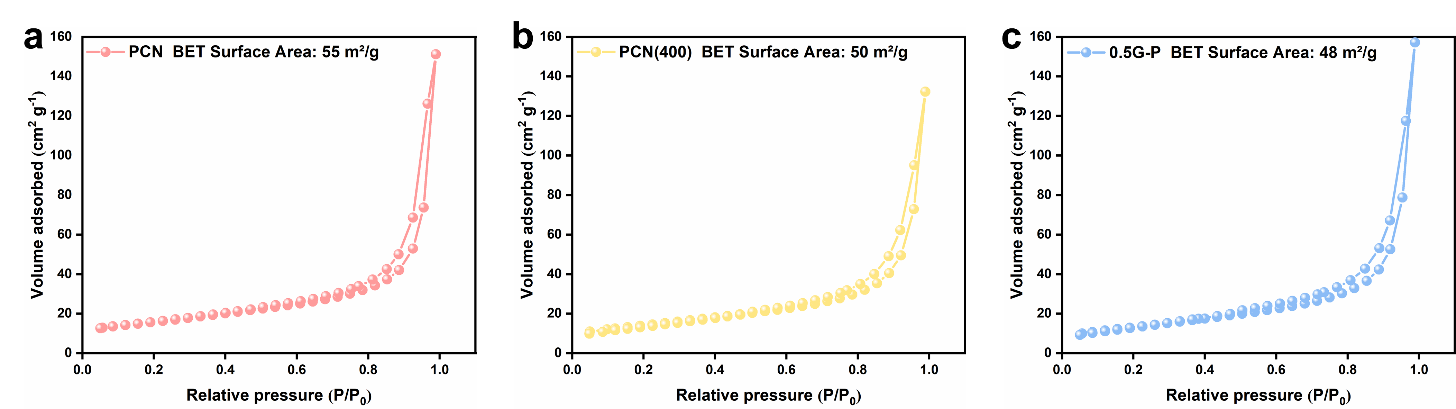


**Figure S3.** The N_2_ adsorption-desorption isotherms of (a) PCN, (b) PCN(400), and (c) 0.5G-P.

All catalysts exhibited Type III isotherms in their BET analysis. Furthermore, the specific surface areas of the catalysts subjected to secondary calcination (PCN(400), 50 m^2^/g) and subsequent compositing with GDY (0.5G-P, 48 m^2^/g) were slightly reduced compared to that of the pristine PCN (PCN, 55 m^2^/g).


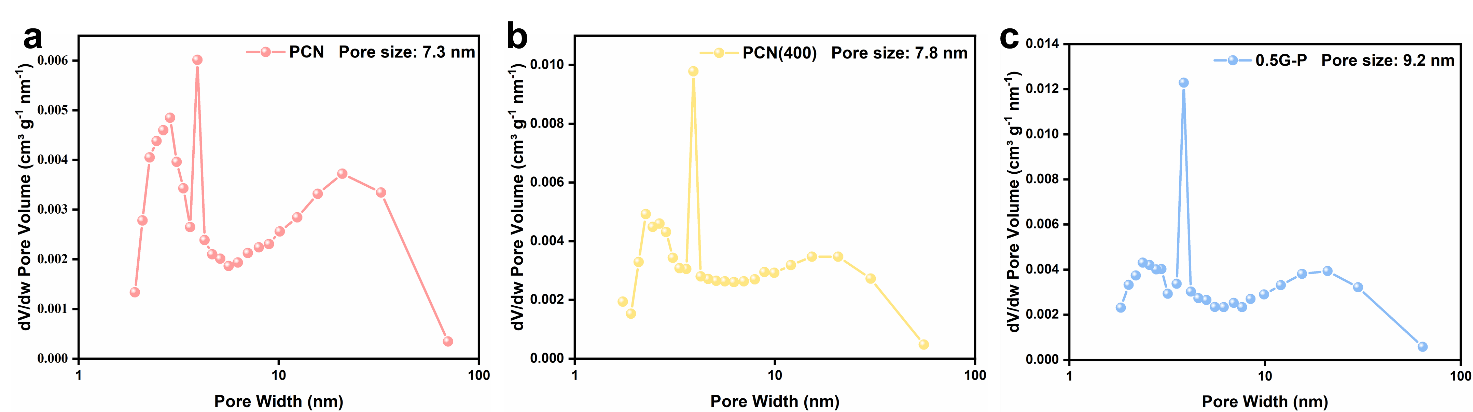


**Figure S4.** The pore size distributions of (a) PCN, (b) PCN(400), and (c) 0.5G-P.

All catalysts exhibited a mesoporous structure. The incorporation of GDY led to a slight increase in the pore size of the resulting catalyst.


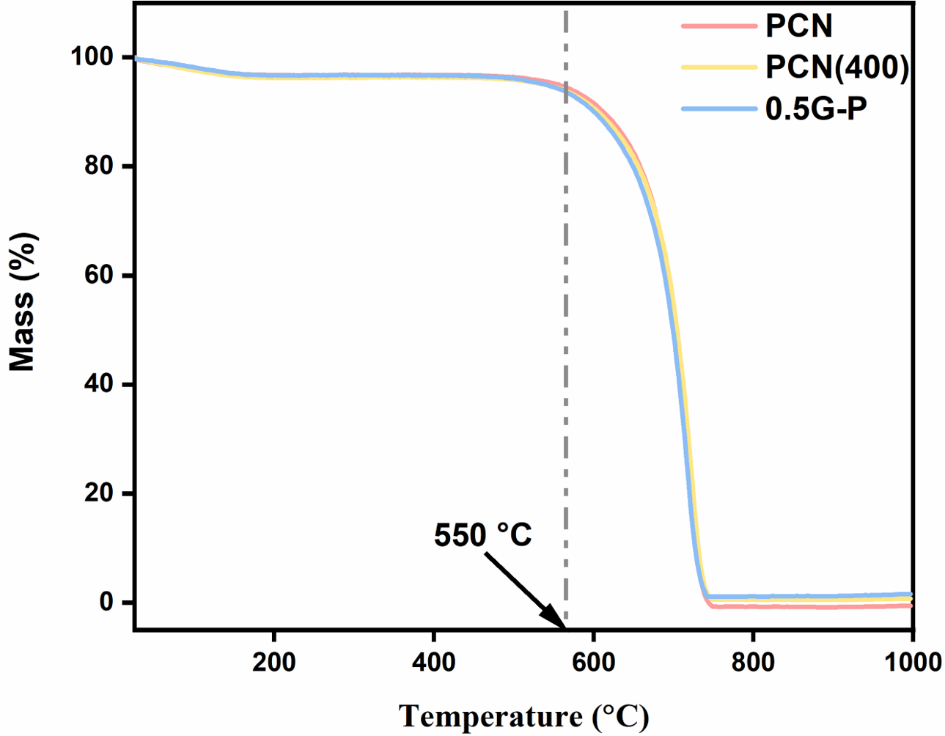


**Figure S5.** The thermogravimetric analysis of the samples.

All samples remained stable at temperatures below 550 °C.


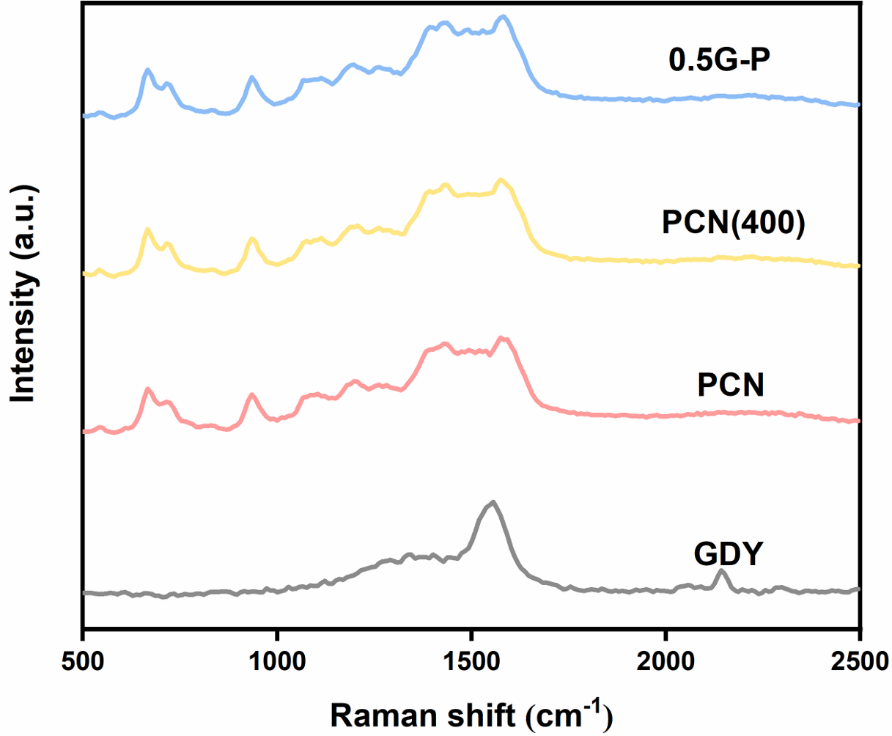


**Figure S6.** Raman spectra of GDY, PCN, PCN(400), and 0.5G-P.

The Raman spectrum of graphdiyne exhibits four prominent peaks. The peak at 1382 cm^-1^ is attributed to the breathing vibrations of sp^2^-hybridized carbon domains within the aromatic rings. The peak at 1570 cm^–1^ associated with the in-phase stretching vibrations of sp^2^ carbon domains in the aromatic rings. The peaks observed at 2190 cm^–1^ and 1926 cm^–1^ can be assigned to the vibrations of conjugated diyne linkages (–C≡C–C≡C–). The Raman spectra of PCN, PCN(400), and 0.5G-P showcase characteristic signals associated with carbon nitride, featuring notable peaks at 708, 990, 1220–1240, and 1550–1580 cm^–1^, which correspond to breathing vibration of heptazine rings, stretching vibration of heptazine skeleton, stretching vibrations of C–N heterocyclic, and in-plane stretching of G band analogue, respectively.


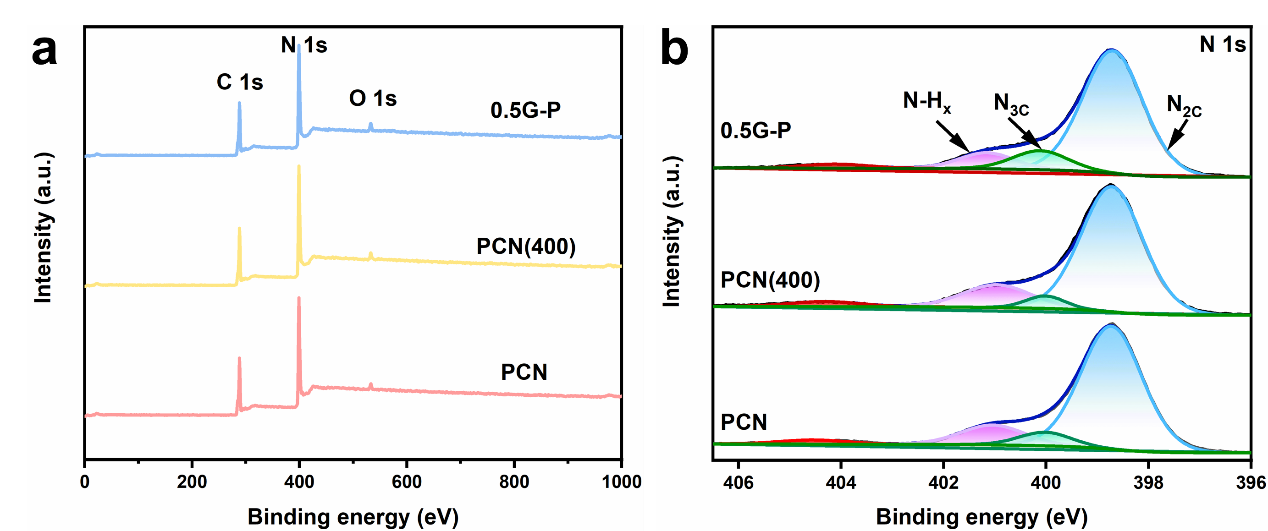


**Figure S7.** (a) The survey XPS spectra of the samples. (b) XPS spectra for N 1s orbitals of the samples.

All catalysts were composed of three elements: C, N, and O. The N 1s XPS spectra for all samples could be deconvoluted into four peaks, corresponding to N–H_x_, N_3C_, N_2C_, and a minor peak attributed to free amino groups (**Figure S7b**).^[12]^


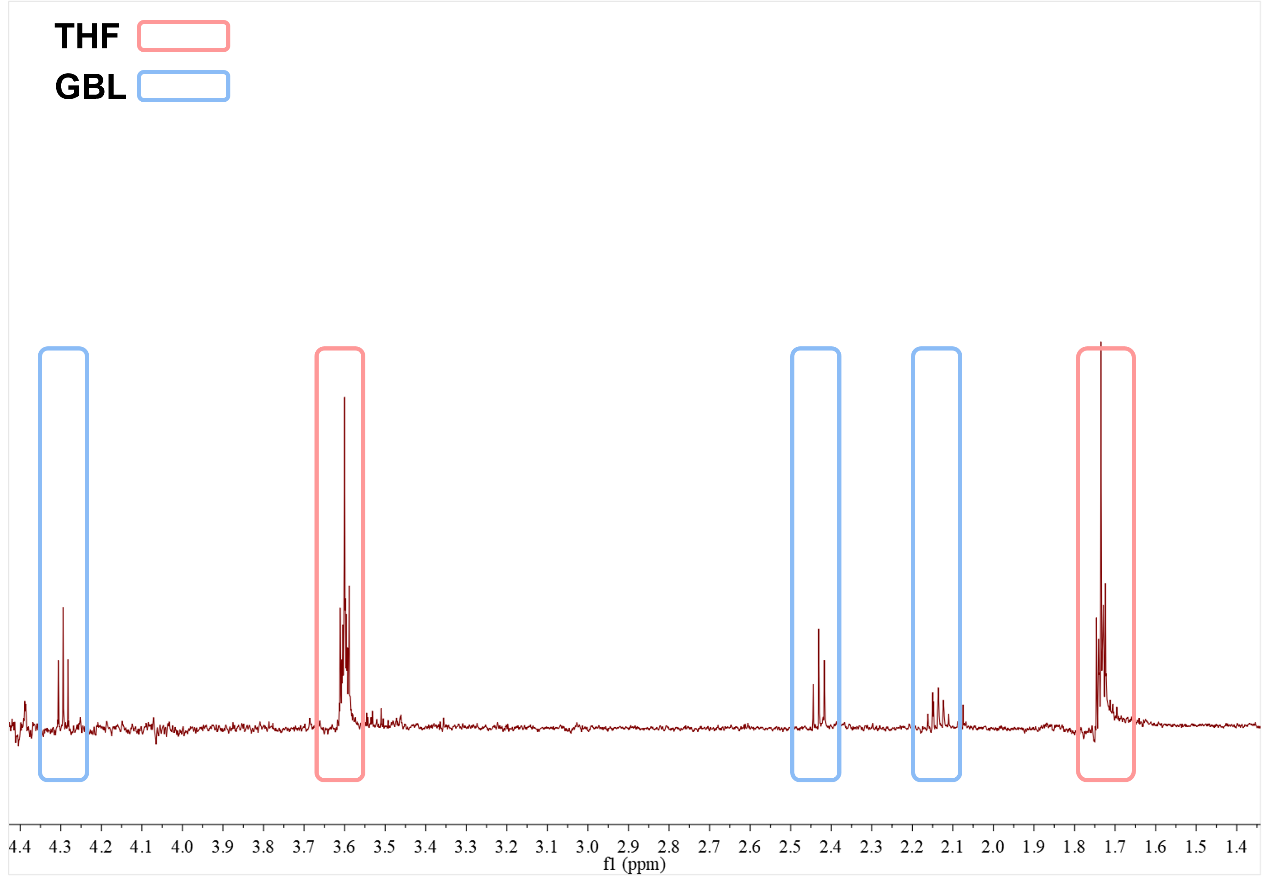


**Figure S8.** ^1^H NMR spectrum of the solution after the reaction.


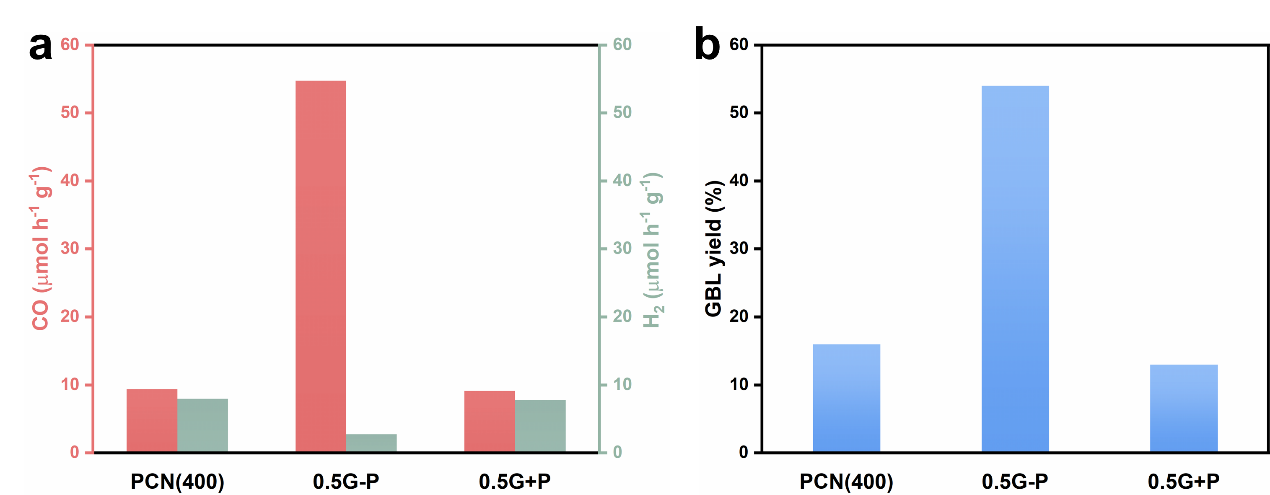


**Figure S9.** (a) CO and H_2_ production rate over different photocatalysts. (b) GBL yield over different photocatalysts. 0.5G+P is a mixture prepared by simply grinding 1 mg of GDY with 200 mg of PCN(400) as a physical blend.


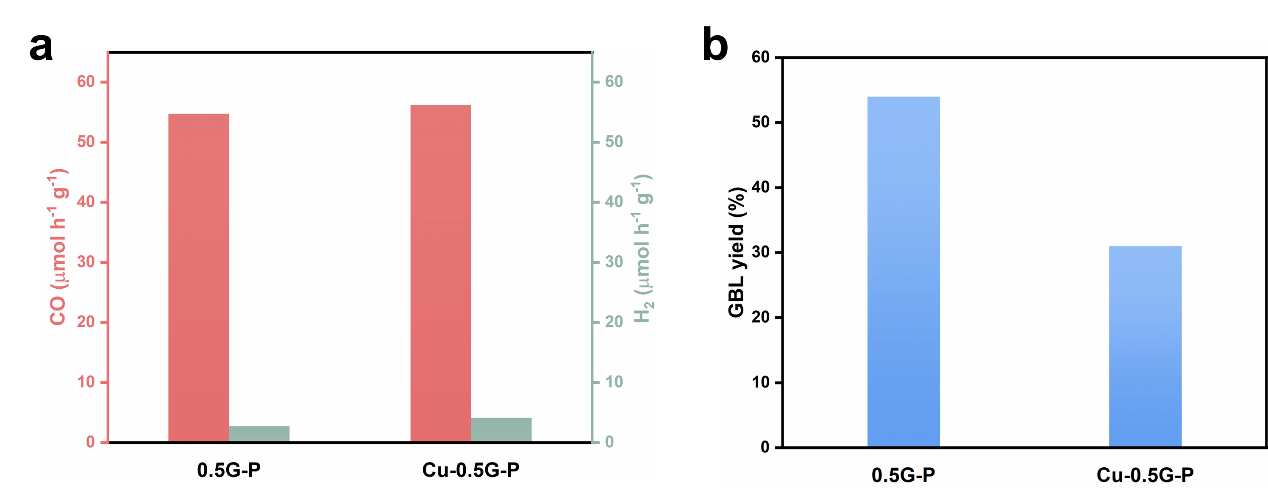


**Figure S10.** (a) CO and H_2_ production rate over different photocatalysts. (b) GBL yield over different photocatalysts. Cu-0.5G-P was synthesized by loading 0.5 wt% copper onto 0.5G-P via a photodeposition method using Cu(NO_3_)_2_·6H_2_O as precursor.


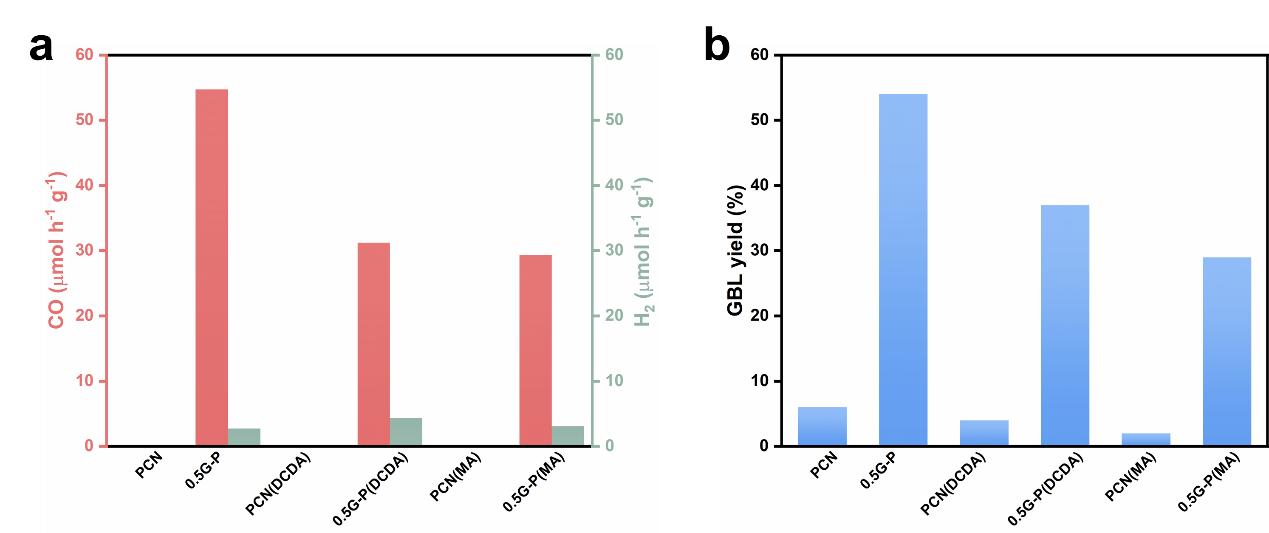


**Figure S11.** (a) CO and H_2_ production rate over different photocatalysts. (b) GBL yield over different photocatalysts. PCN(DCDA) and PCN(MA) were separately synthesized using dicyandiamide (DCDA) and melamine (MA) as precursors, and then composited with GDY to obtain 0.5G-P(DCDA) and 0.5G-P(MA), respectively.


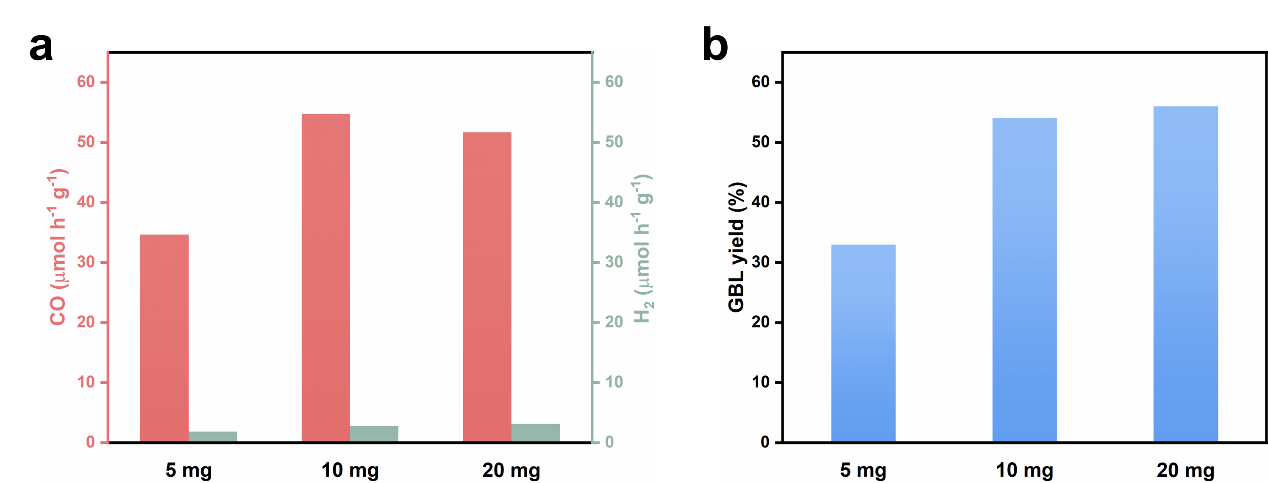


**Figure S12.** (a) CO and H_2_ production rate over and (b) GBL yield using different amounts of catalyst.

The photocatalytic activity was low when the catalyst dosage was 5 mg. Increasing the dosage to 10 mg led to a significant enhancement in activity. However, when the dosage was further increased to 20 mg, no substantial improvement in activity was observed. In theory, a higher catalyst loading provides more active sites, which would be expected to enhance activity. Nevertheless, with a fixed volume of water (2 mL), increasing the catalyst dosage inevitably raises its concentration in the reaction system. Such an increase in catalyst concentration may induce a light-shielding effect, thereby compromising the overall activity.


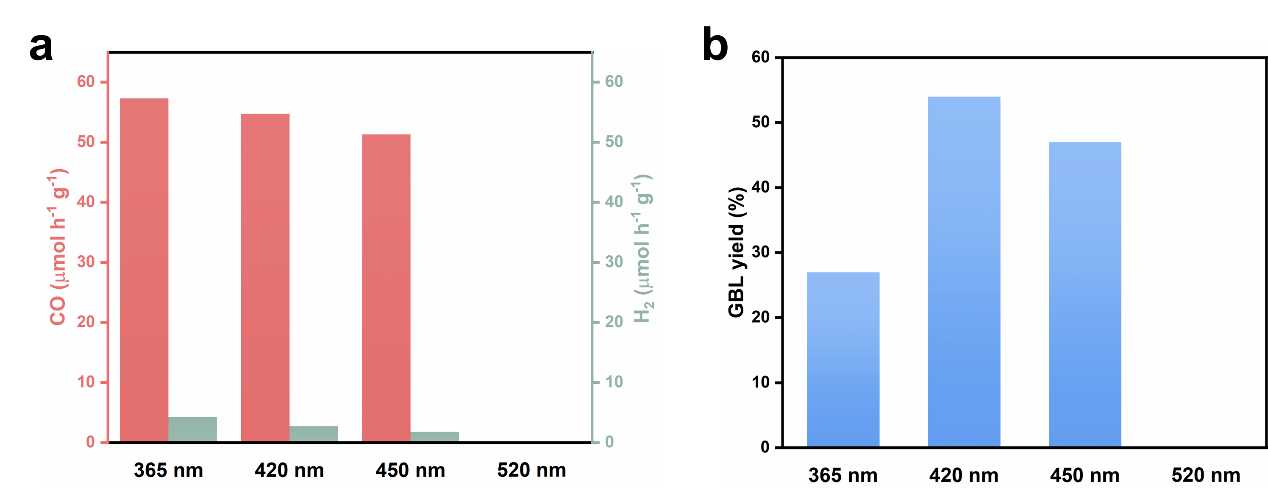


**Figure S13.** (a) CO and H_2_ production rate and (b) GBL yield under different LED wavelength illumination.

The results show that at 365 nm, the CO yield of 0.5G-P is slightly enhanced, but the GBL yield decreases. Notably, a significant increase in ring-opening products is observed under 365 nm irradiation, which is likely attributable to the high energy of ultraviolet light. The activity at 450 nm is slightly lower than that at 420 nm. No redox products were detected at 520 nm, as the catalyst does not absorb light at this wavelength.

**
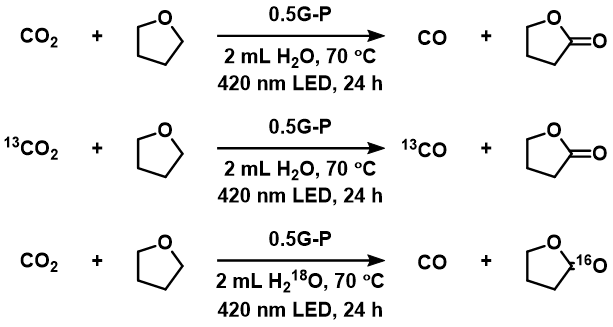
**

**Figure S14.** Isotopic labeling with ^13^CO_2_ and H_2_^18^O.


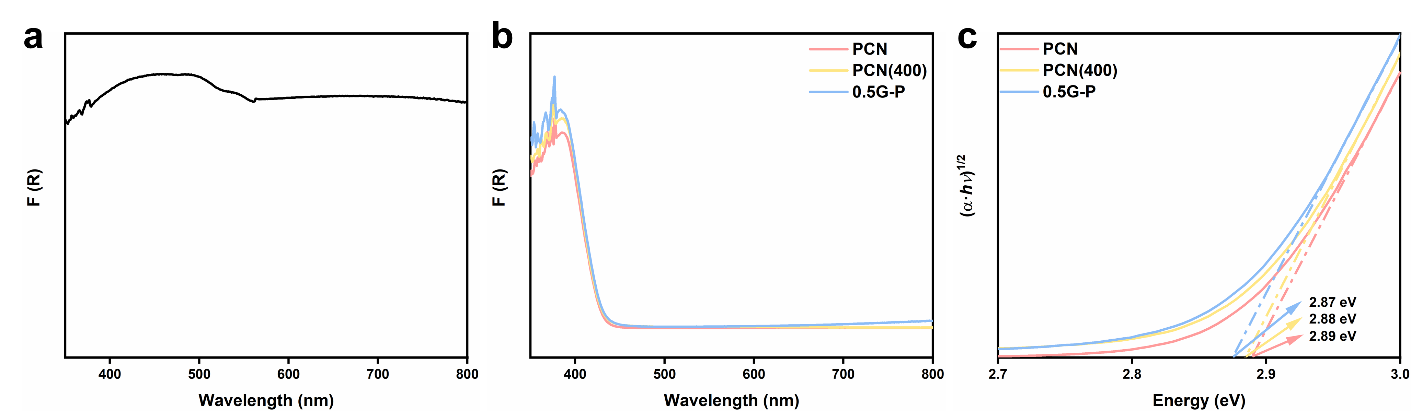


**Figure S15.** Diffuse reflectance spectra of (a) GDY and (b) Photocatalysts. (c) Kubelka-Munk plot of photocatalysts.

Due to its narrow band gap, GDY exhibits full-spectrum absorption within the visible light range. The three photocatalysts displayed similar band gap energies.


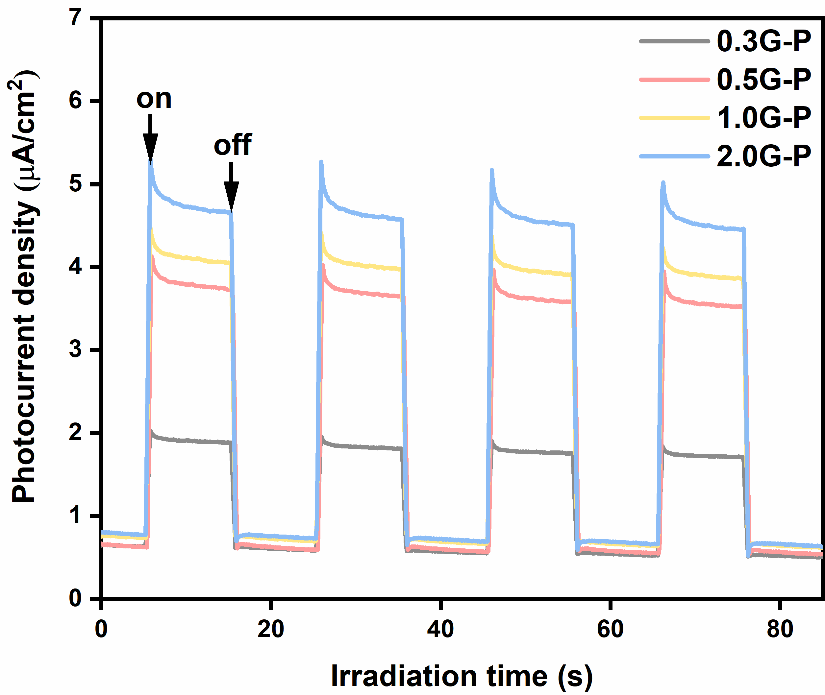


**Figure S16.** Photocurrent response of samples with varying GDY contents.


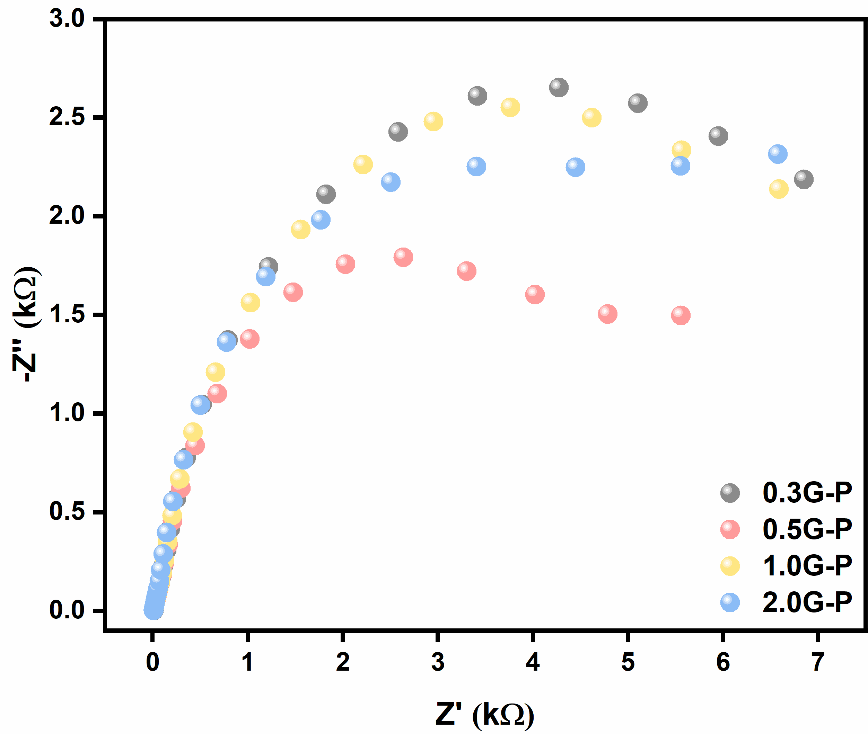


**Figure S17.** EIS plots of samples with varying GDY contents.


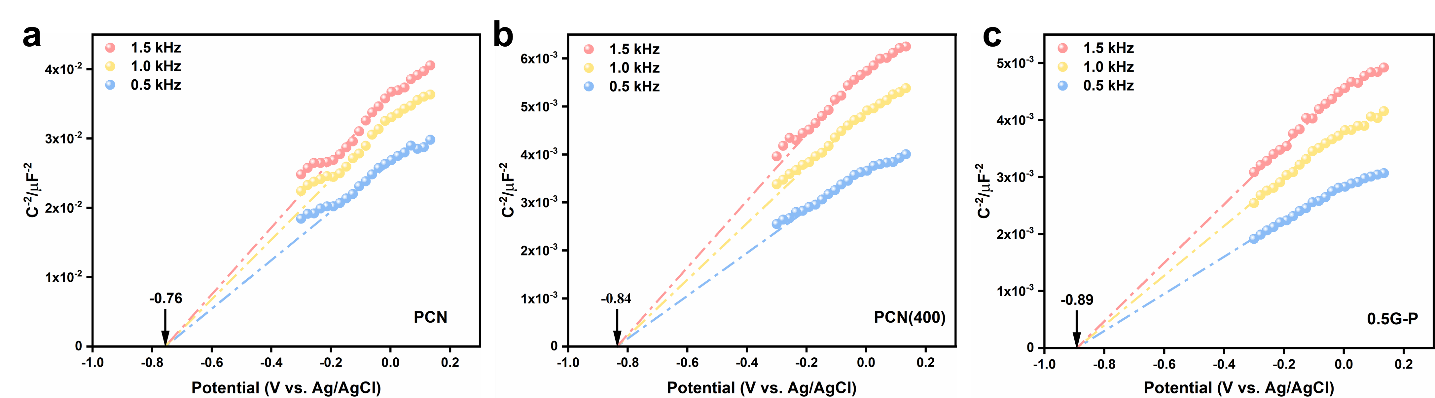


**Figure S18.** Mott-Schottky plots of the catalysts.

The conduction band potentials of all catalysts satisfied the thermodynamic requirements for the reduction of CO_2_ to CO.


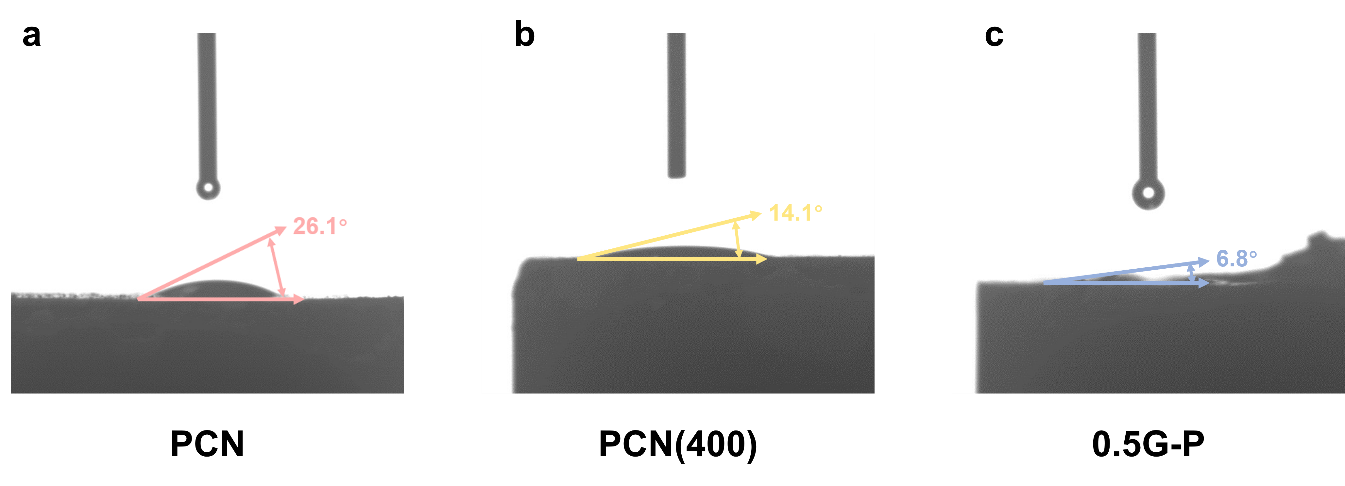


**Figure S19.** Contact angle test of (a) PCN, (b) PCN(400), and (c) 0.5G-P.

The pristine PCN exhibited a water contact angle of 26.1°. PCN(400) displayed enhanced hydrophilicity with a contact angle of 14.1°. Following compositing with graphdiyne, 0.5G-P became highly hydrophilic, characterized by a contact angle of only 6.8°. This markedly increased hydrophilicity facilitates its more uniform dispersion in aqueous reaction media and promotes more effective contact with reactants.

**
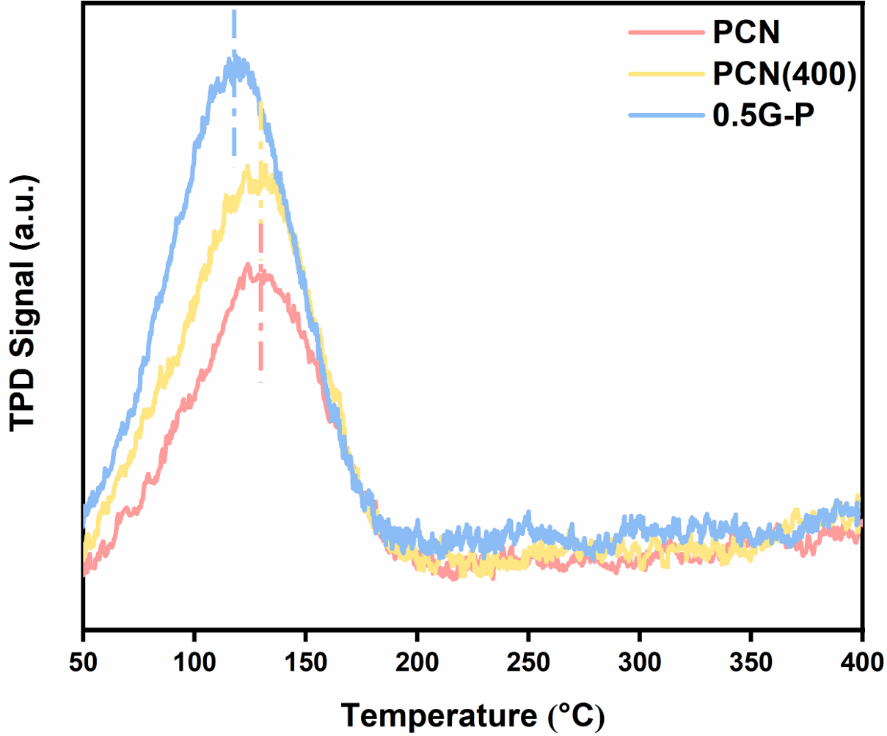
**

**Figure S20.** CO_2_ temperature-programmed desorption profiles of samples.


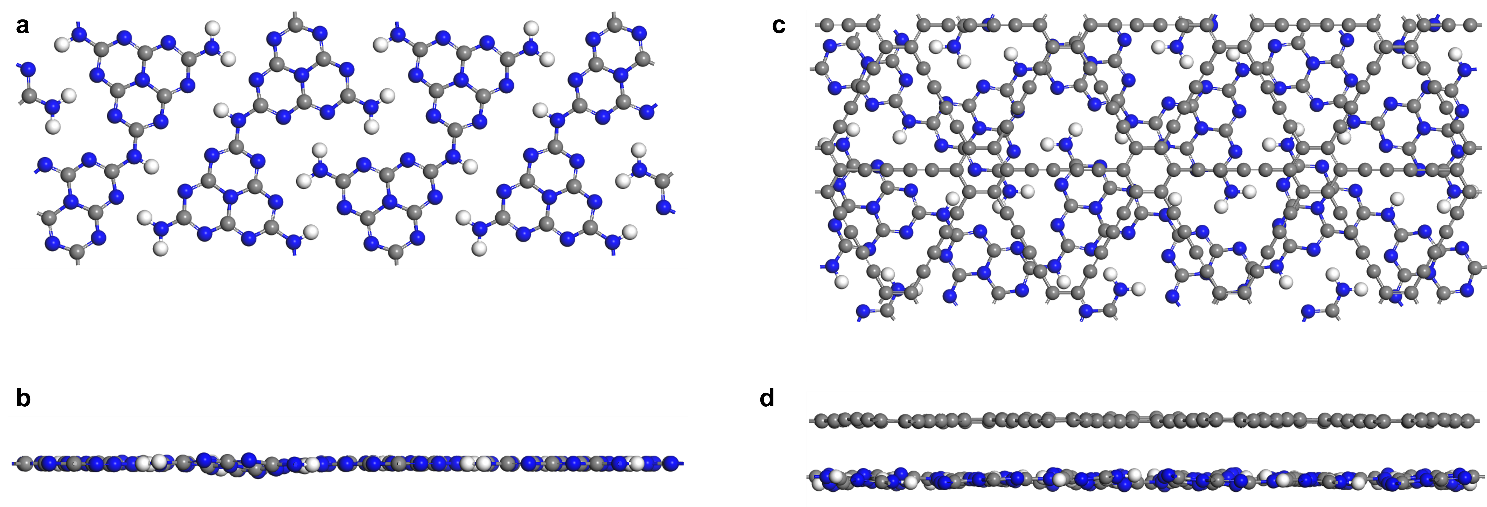


**Figure S21.** PCN models (a and b) used for DFT calculations; G-P models (c and d).


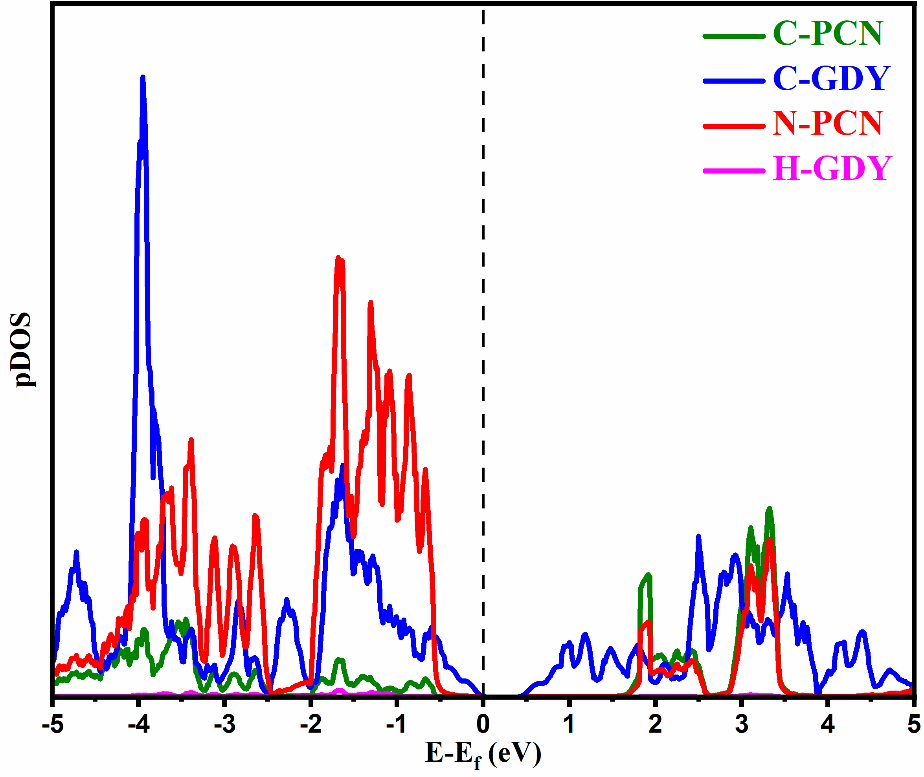


**Figure S22.** pDOS plot of the G-P model. The colored curves represent the orbital contributions of different elements originating from GDY and PCN.


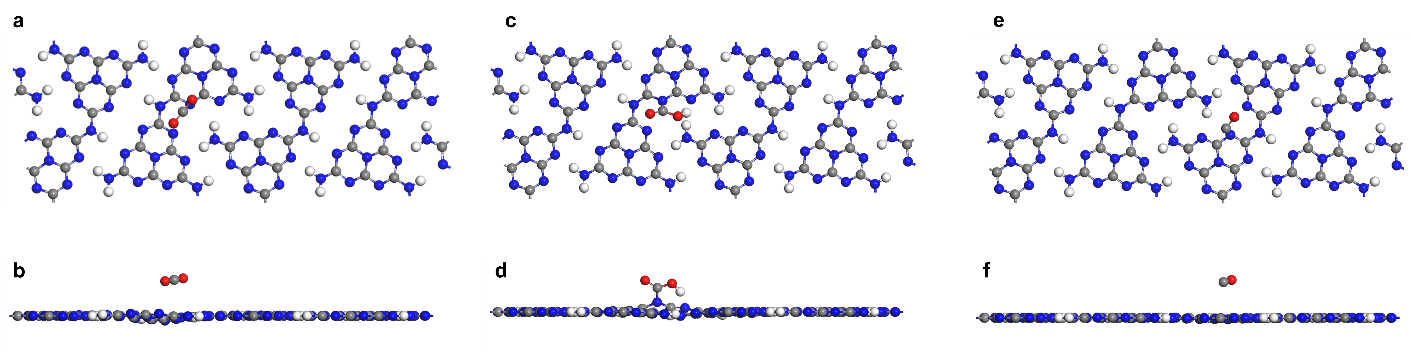


**Figure S23.** Model images of CO_2_* (a and b), COOH* (c and d), and CO* (e and f) over PCN.


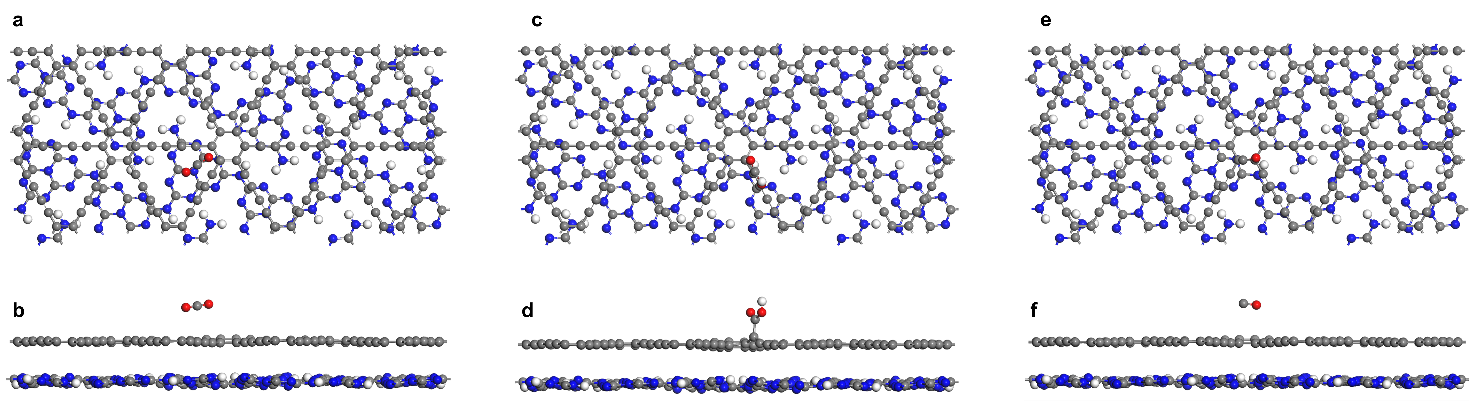


**Figure S24.** Model images of CO_2_* (a and b), COOH* (c and d), and CO* (e and f) over G-P.


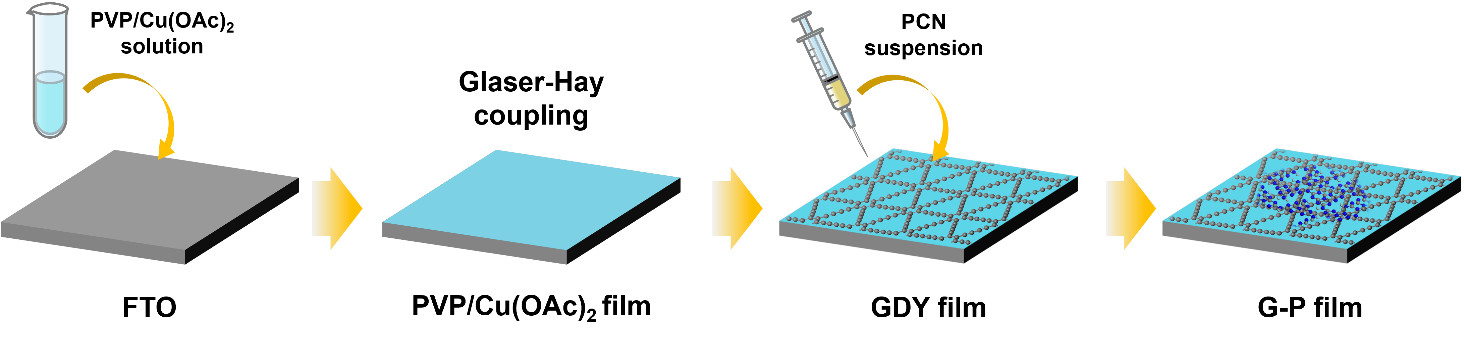


**Figure S25.** Schematic diagram of the deposition of GDY film and PCN nanosheets on FTO.

GDY film was synthesized according to a previous method.^[13]^ 0.3 g of PVP and 0.4 g of cupric acetate was dissolved in 20 mL deionized water to get a slow-release solution with Cu^2+^ concentration. 15 μL of as prepared PVP/Cu(OAc)_2_ solution was dropped on the FTO glass, followed by spinning at 3000 rpm to fabricate a PVP/Cu(OAc)_2_ film. For the powder substrates, the substrates were immersed in PVP/Cu(OAc)_2_ solution for 3 hours, then filtered and dried to form a PVP/Cu(OAc)_2_ film. The substrates covered with PVP/Cu(OAc)_2_ film was immersed into HEB solution with acetone and pyridine (v: v = 20: 1) as solvent, followed by keeping 3 days’ standing in atmosphere environment at room temperature. Finally, bulk GDY was uniformly fabricated on the target substrates.

Separately, 200 mg of PCN was sonicated in 100 mL of deionized water for 2 h. Subsequently, 150 μL of as prepared PCN solution was dropped on the GDY film, followed by spinning at 3000 rpm. the resulting film was heated at 400 ℃ for 2 h under a nitrogen atmosphere with a heating rate of 4 ℃ min^-1^.


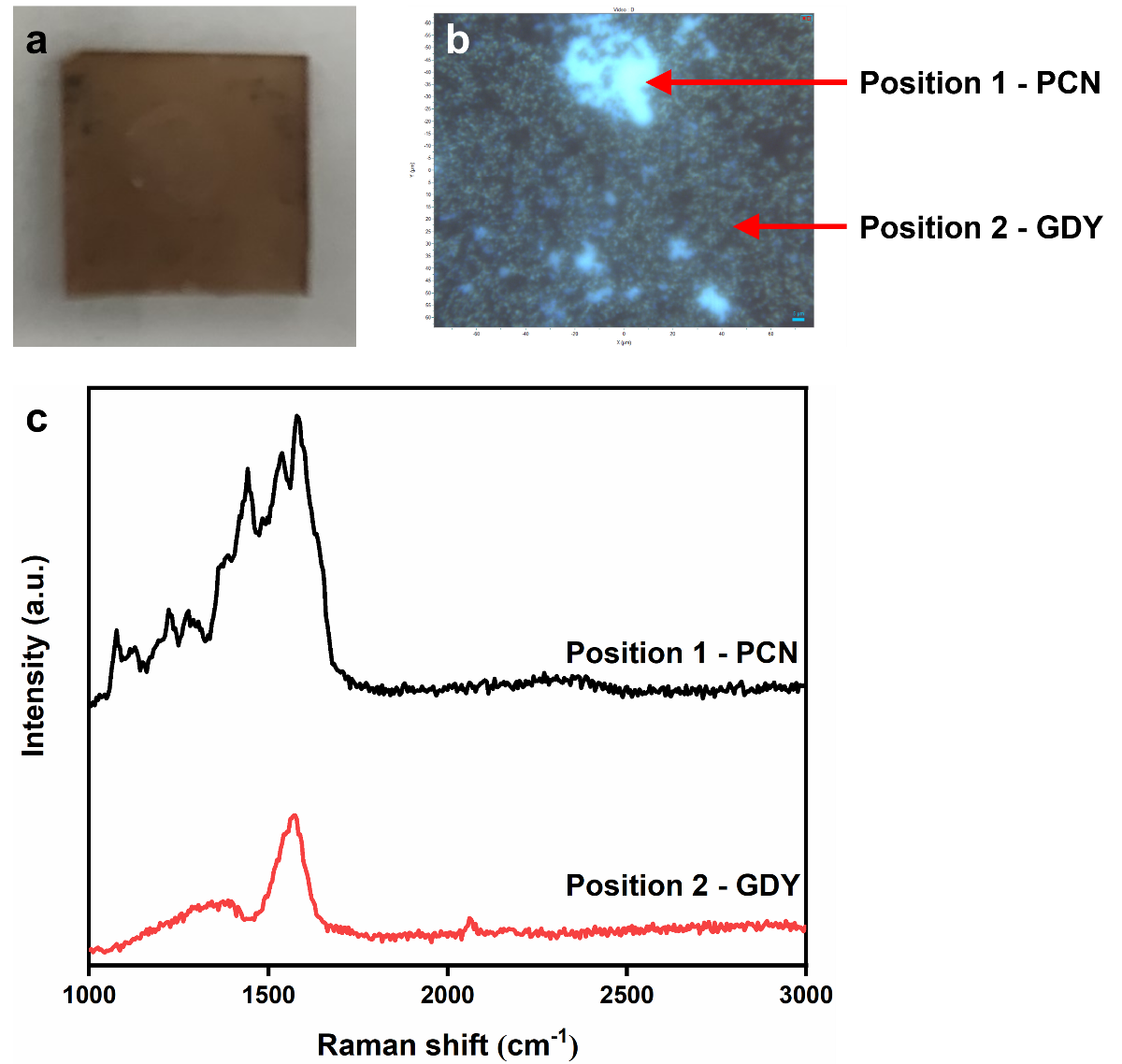


**Figure S26.** (a) Photograph of the PCN-GDY film grown on FTO. (b) Photograph of the film under an optical microscope attached to a Raman spectrometer, showing white PCN nanosheets and the dark GDY film. (c) Raman spectra acquired at positions 1 and 2. The PCN nanosheets supported on the uniform GDY film surface can be clearly identified using Raman spectroscopy in conjunction with optical microscopy.


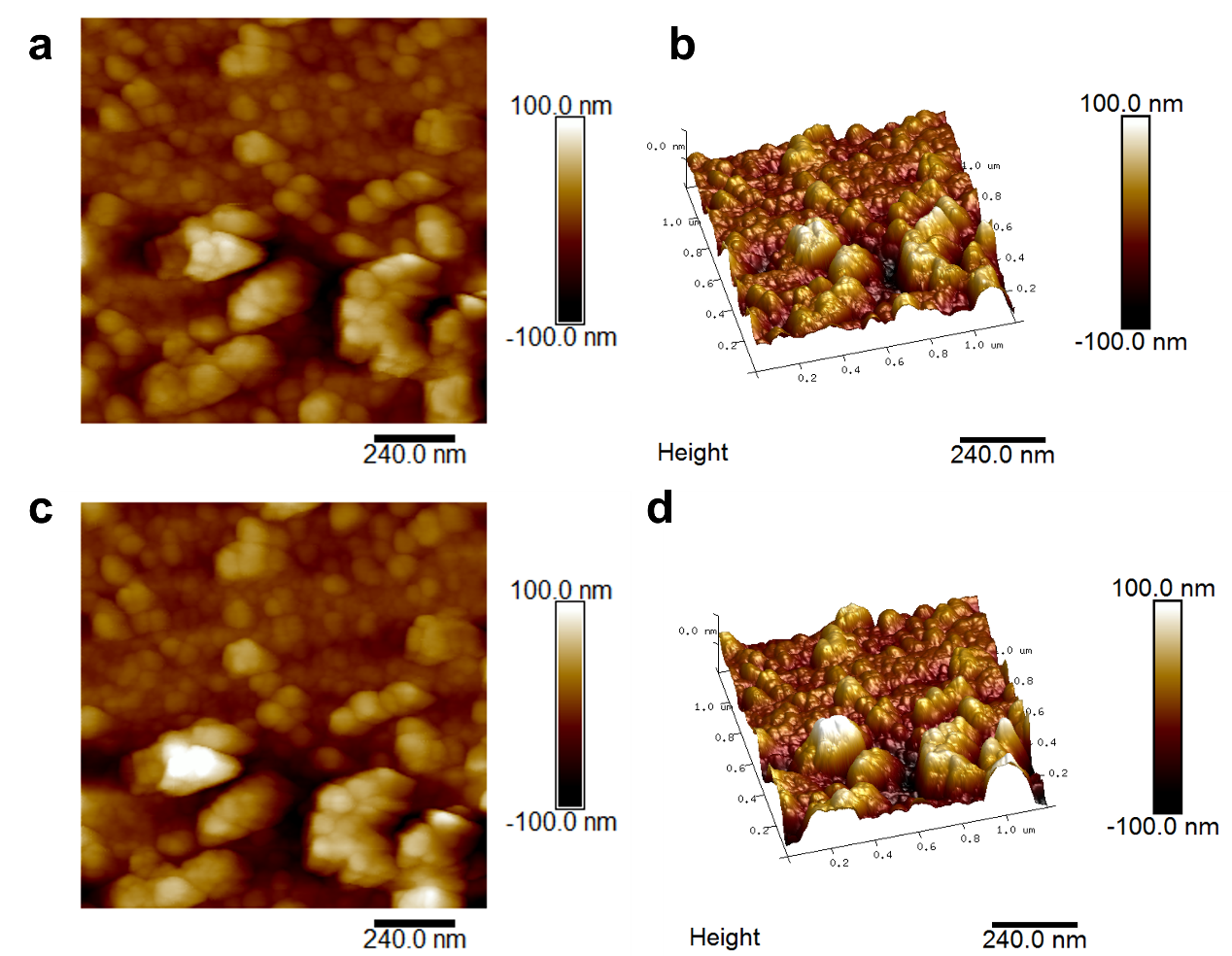


**Figure S27.** AFM height images of the scanned area before illumination: (a) 2D, (b) 3D. AFM height images of the scanned area under illumination: (c) 2D, (d) 3D.





**Figure S28.** The EPR spectra of the samples under irradiation (50 W, 420 nm LED).


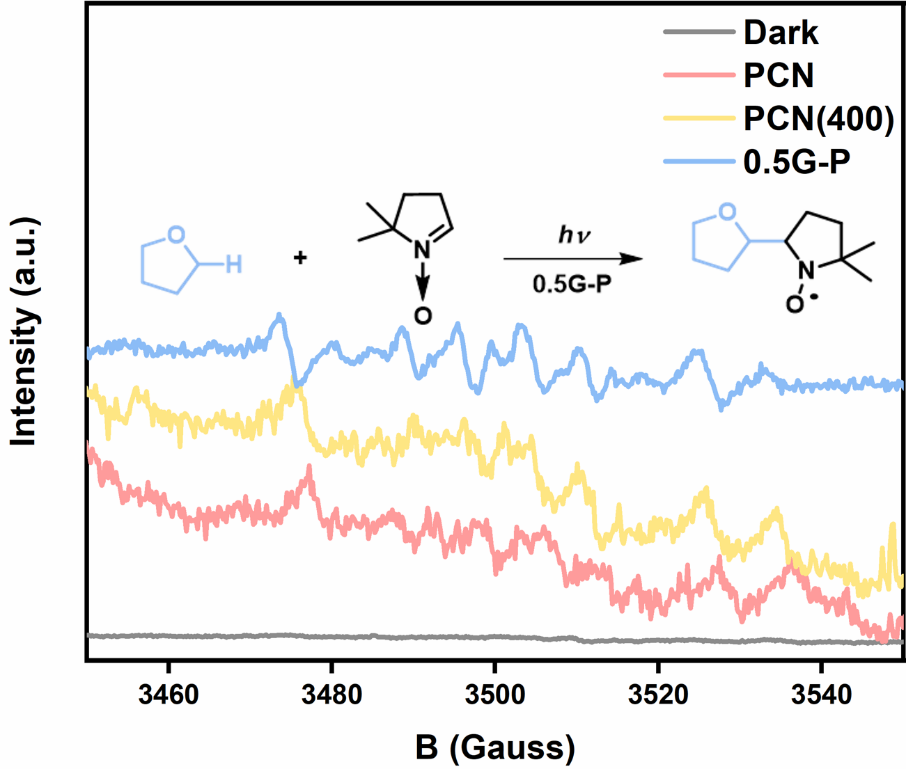


**Figure S29.** Characteristic EPR signal of the carbon radical trapped by DMPO.

No EPR signal was observed in the dark. Under illumination, only weak radical signals were detected for PCN and PCN(400), whereas a stable and prominent carbon radical signal was observed for 0.5G-P.^[14]^


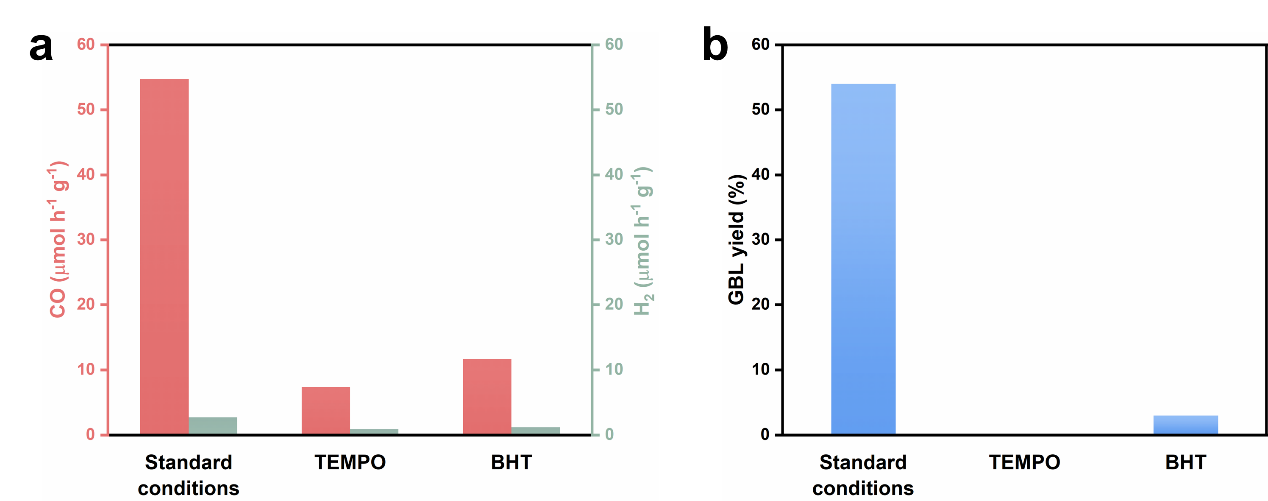


**Figure S30.** (a) CO and H_2_ production rate and (b) GBL yield using different radical scavengers.

**Table S1.** A comparison of photocatalytic performance of 0.5G-P with reported photocatalysts.

| **Sample** | **Reaction conditions** | **CO yield**  **(μmol h^-1^ g^-1^)** | **CO Sel.** | **Reference** |
| --- | --- | --- | --- | --- |
| 0.5G-P | 50 W LED (420 nm) , 10 mg, 70 °C, CO_2_, 2 mL H_2_O, 0.25 mmol THF | 55 | 95 | This work |
| FLI2/CNNS-2 | 300 W Xe lamp (320 nm-780 nm), CO_2_, 3 mL H_2_O | 34 | - | ^[15]^ |
| S-CN | 300 W Xe lamp, 50 mg, CO_2_, 100 mL H_2_O, 25 °C | 16 | - | ^[16]^ |
| V_N_-PCN | 300 W Xe lamp (320–780 nm), 20 mg, CO_2_ (70 kPa), 2 mL H_2_O | 16 | - | ^[17]^ |
| CQDs/g-C3N4 | 300 W Xe-lamp (>420 nm), 100 mg, CO_2_,10 mL H_2_O, 20 mL MeCN, 20 mL TEOA | 19 | - | ^[18]^ |
| KP/CN-2 | 300 W Xe lamp (λ>420 nm), 10 mg, 80 kPa CO_2_, 0.5 mL H_2_O, | 12 | 67 | ^[19]^ |
| g-C_3_N_4_ NSs | 300 W Xenon lamp, 2 mg, CO_2_, 1 mL H_2_O | 2 | 86 | ^[20]^ |
| KOCN | 300 W Xenon lamp, 30 mg, CO_2_, 500 μL H_2_O | 18 | 93 | ^[21]^ |
| ACNNT | 300 W Xenon lamp (>420 nm), 25 mg, CO_2_, 6 mL MeCN, 4 mL H_2_O, 2 mL TEOA | 88 | ~90 | ^[22]^ |
| MTCN | 35 W Xenon lamp (>420 nm), 10 mg, CO_2_ | 8 | - | ^[23]^ |

**Table S2.** A comparison of photocatalytic performance of 0.5G-P with PCN modified with conventional noble metal cocatalysts


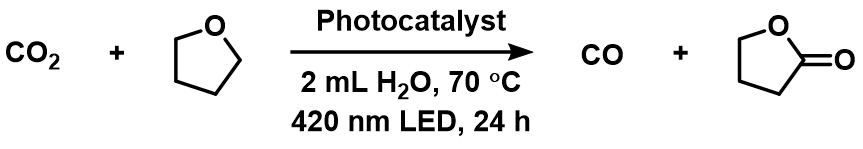


| **Entry** | **Photocatalyst** | **CO (μmol h^-1^ g^-1^)** | **CO Sel.**  **(%)** | **GBL Yield**  **(%)** | **GBL Sel.**  **(%)** |
| --- | --- | --- | --- | --- | --- |
| 1 | 0.5G-P | 55 | 95 | 54 | >99 |
| 2 | Cu-PCN | 23 | 12 | 14 | 78 |
| 3 | Co-PCN | 31 | 43 | 7 | 26 |
| 4 | Pt-PCN | 14 | 7 | 22 | 64 |

**Table S3.** Control experiments.


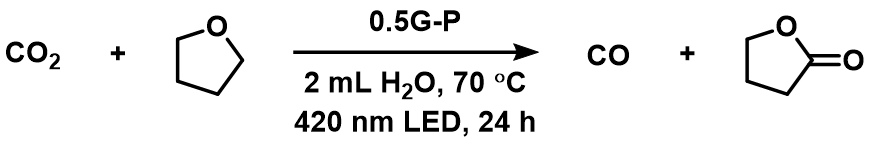


| **Entry** | **Photocatalyst** | **CO (μmol h^-1^ g^-1^)** | **CO Sel.**  **(%)** | **GBL Yield (%)** | **GBL Sel.**  **(%)** |
| --- | --- | --- | --- | --- | --- |
| 1 | 0.5G-P | 55 | 95 | 54 | >99 |
| 2^[a]^ | 0.5G-P | N.D. | - | N.D. | - |
| 3^[b]^ | 0.5G-P | N.D. | - | N.D. | - |

[a] Without catalyst during reaction. [b] Without light irradiation during the reaction.

**Table S4.** Mass of catalyst after reaction.


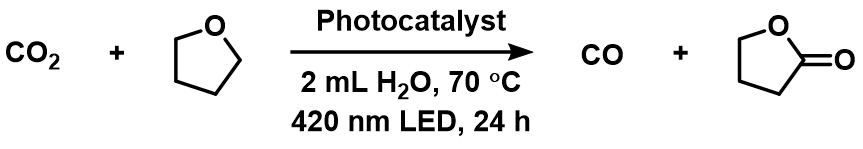


| **Cycle** | **Mass of catalyst after reaction (mg)** |
| --- | --- |
| 1 | 8.2 |
| 2 | 7.6 |
| 3 | 7.3 |

**Reference**

[1] G. Li, Y. Li, H. Liu, Y. Guo, Y. Li, D. Zhu, *Chem. Commun.* **2010**, 46, 3256–3258.

[2] Y. Wang, J. Sun, Y. Yao, Z. Li, X. Meng, *Int. J. Hydrogen Energy* **2022**, 47, 28007–28018.

[3] G. Kresse, D. Joubert, *Phys. Rev. B* **1999**, 59, 1758-1775.

[4] P. E. Blöchl, *Phys. Rev. B* **1994**, 50, 17953-17979.

[5] J. P. Perdew, K. Burke, M. Ernzerhof, *Phys. Rev. Lett.* **1996**, 77, 3865-3868.

[6] S. Grimme, S. Ehrlich, L. Goerigk, *J. Comput. Chem.* **2011**, 32, 1456-1465.

[7] V. Wang, N. Xu, J.-C. Liu, G. Tang, W.-T. Geng, *Comput. Phys. Commun.* **2021,** 267, 108033.

[8] J. K. Nørskov, J. Rossmeisl, A. Logadottir, L. Lindqvist, J. R. Kitchin, T. Bligaard, H. Jónsson, *J. Phys. Chem. B* **2004**, 108, 17886-17892.

[9] J. K. Nørskov, T. Bligaard, A. Logadottir, J. R. Kitchin, J. G. Chen, S. Pandelov, U. Stimming, *J. Electrochem. Soc.* **2005**, 152, J23.

[10] P. Yadav, R. Pratap, V. Ji Ram, *Asian J. Org. Chem.* **2020**, 9, 1377–1409.

[11] B. Mao, M. Fañanás-Mastral, B. L. Feringa, *Chem. Rev.* **2017**, 117, 15, 10502–10566

[12] G. Xiong, Z. Zhang, Y. Qi, Nanomaterials **2022**, 12.

[13] F. Zhao, N. Wang, M. Zhang, A. Sápi, J. Yu, X. Li, W. Cui, Z. Yang, C. Huang, *Chem. Commun.* **2018**, 54, 6004–6007.

[14] J. Qiao, Z. Q. Song, C. Huang, R. N. Ci, Z. Liu, B. Chen, C. H. Tung, L. Z. Wu, *Angew. Chem. Int. Ed.* **2021**, 60, 27201–27205.

[15] H. Liu, S. Cao, L. Chen, K. Zhao, C. Wang, M. Li, S. Shen, W. Wang, L. Ge, *Chem. Eng. J.* **2022**, 433, 133594.

[16] Z. Zhu, Z. Liu, X. Tang, K. Reeti, P. Huo, J. W. C. Wong, J. Zhao, Catal. Sci. *Technol.* **2021**, 11, 1725–1736.

[17] D. Zeng, X. Wang, Y. Liu, D. Liu, Z. Zhang, L. Fei, J. Robertson, C. Kuai, Y. Guo, *ACS Sustain. Chem. Eng.* **2022**, 10, 5758–5769.

[18] Z. Liu, W. Hou, H. Guo, Z. Wang, L. Wang, M. Wu, *ACS Appl. Mater. Interfaces* **2023**, 15, 33868–33877.

[19] M. Chen, M. Guo, M. Zhai, J. Xu, L. Wang, *J. CO_2_ Util.* **2023**, 68, 102392.

[20] X. Jing, X. Mi, W. Lu, N. Lu, S. Du, G. Wang, Z. Zhang, *Chin. J. Catal.* **2024**, 67, 112–123.

[21] C. Guan, Y. Liao, Q. Xiang, *Sci. China Mater.* **2024**, 67, 473–483.

[22] L. Chen, H. Li, H. Li, H. Li, W. Qi, Q. Zhang, J. Zhu, P. Zhao, S. Yang, *Appl. Catal. B* **2022**, 318, 121863.

[23] Y. Yang, Y. Chen, Z. Li, S. Tang, Y. Li, Z. Fu, S. Yang, M. Yang, H. Xie, *Chem. Eng. J.* **2022**, 430, 132668.
